# Novel Tumor-Specific Antigens for Immunotherapy Identified From Multi-omics Profiling in Thymic Carcinomas

**DOI:** 10.3389/fimmu.2021.748820

**Published:** 2021-11-16

**Authors:** Wentao Fang, Chia-Hsin Wu, Qiang-Ling Sun, Zhi-Tao Gu, Lei Zhu, Teng Mao, Xue-Fei Zhang, Ning Xu, Tzu-Pin Lu, Mong-Hsun Tsai, Li-Han Chen, Liang-Chuan Lai, Eric Y. Chuang

**Affiliations:** ^1^ Department of Thoracic Surgery, Shanghai Chest Hospital, Shanghai JiaoTong University, Shanghai, China; ^2^ Graduate Institute of Biomedical Electronics and Bioinformatics, National Taiwan University, Taipei, Taiwan; ^3^ Thoracic Cancer institute, Shanghai Chest Hospital, Shanghai Jiao Tong University, Shanghai, China; ^4^ Department of Pathology, Shanghai Chest Hospital, Shanghai Jiao Tong University, Shanghai, China; ^5^ Bioinformatics and Biostatistics Core, Centers of Genomic and Precision Medicine, National Taiwan University, Taipei, Taiwan; ^6^ Department of Public Health, National Taiwan University, Taipei, Taiwan; ^7^ Institute of Biotechnology, National Taiwan University, Taipei, Taiwan; ^8^ Institute of Fisheries Science, National Taiwan University, Taipei, Taiwan; ^9^ Graduate Institute of Physiology, College of Medicine, National Taiwan University, Taipei, Taiwan; ^10^ Department of Electrical Engineering, National Taiwan University, Taipei, Taiwan; ^11^ Master Program for Biomedical Engineering, China Medical University, Taichung, Taiwan

**Keywords:** thymic carcinoma (TC), whole-exome sequencing (WES), RNA sequencing (RNAseq), immunotherapy, neoantigen, immune checkpoint inhibitor (ICI), gene fusion, driver alteration

## Abstract

Thymic carcinoma (TC) is the most aggressive thymic epithelial neoplasm. TC patients with microsatellite instability, whole-genome doubling, or alternative tumor-specific antigens from gene fusion are most likely to benefit from immunotherapies. However, due to the rarity of this disease, how to prioritize the putative biomarkers and what constitutes an optimal treatment regimen remains largely unknown. Therefore, we integrated genomic and transcriptomic analyses from TC patients and revealed that frameshift indels in *KMT2C* and *CYLD* frequently produce neoantigens. Moreover, a median of 3 fusion-derived neoantigens was predicted across affected patients, especially the *CATSPERB*-*TC2N* neoantigens that were recurrently predicted in TC patients. Lastly, potentially actionable alterations with early levels of evidence were uncovered and could be used for designing clinical trials. In summary, this study shed light on our understanding of tumorigenesis and presented new avenues for molecular characterization and immunotherapy in TC.

## 1 Introduction

Thymic carcinoma (TC) is an extremely rare malignancy worldwide and constitutes just a minority of thymic epithelial tumors (TETs) (1). According to the World Health Organization (WHO) histologic grade classification, TCs are poorly differentiated TETs, harboring a more aggressive clinical course than thymomas, with a 36% 5-year survival rate (1). To date, surgery is still the recommended treatment for localized TCs, while chemotherapy is the standard first-line therapy for advanced and nonresectable TCs (2). However, a majority of these patients will still suffer disease progression after platinum-based chemotherapy, owing to a lack of standard second-line treatments and effective, universal targeted therapies (3). As attempts to develop new targeted therapies have been hampered by the rarity of this disease and our limited understanding of its somatic alterations, the need for uncovering a comprehensive mutational landscape in TC has become apparent.

Accumulating evidence suggests that TCs are infrequently associated with paraneoplastic autoimmune disorders (3) and display high expression of PD-L1 (4), which inhibits the response of cytotoxic T cells by binding to PD-1, thereby down-modulating their antitumor immunity. These findings prompted the use of immune checkpoint inhibitors (ICIs), which block the interaction between CTLA-4, PD-1, and their ligands, as an alternative treatment option in TC. Thus, several clinical trials have begun to assess the tumor response to ICIs in TC patients who had progressive disease after at least one course of chemotherapy (5–7). Two clinical trials of pembrolizumab, a monoclonal antibody targeting PD-1, reported a response rate of 19% and 23%, and progression-free survival of 6.1 and 4.2 months, with a positive correlation between PD-L1 expression and therapeutic efficacy (6, 7). However, another anti-PD-1 antibody (nivolumab) trial showed no response in TC tumors (5). This raises the question of which type of TC most likely triggers antitumor activity upon exposure to ICIs.

Neoantigens, which are presented by specific major histocompatibility complexes (MHCs) and recognized by T cells, also play an important role in immunotherapy (8). The most commonly studied neoantigens for therapeutic vaccines are derived from nonsynonymous single-nucleotide variants (SNVs) (8, 9). However, there are some drawbacks to be noted: (1) most SNV-derived mutated peptides are non-immunogenic, due to their low sequence divergence from the germline source (10, 11); and (2) they are largely patient-specific, making them less likely to be used for off-the-shelf immunotherapies (12, 13). Based on these considerations, alternative tumor-specific antigens (TSAs), a source of immunogenic neoantigens other than SNVs, may mediate more effective and more generalized responses to immunotherapy (8, 11, 14, 15). It has been shown that some cancer types with low tumor mutational burden (TMB) produce more alternative TSAs, which may represent promising targets for immunotherapy, especially with anti-PD-1 treatment (14). Moreover, certain recurrent alternative TSAs may exist across populations or cancer types (8). Therefore, the identification of alternative TSAs may increase the scope of benefit from immunotherapy for TC patients.

Motivated by the importance of TC and its limited treatment options, we performed comprehensive genomic and transcriptomic analyses of 22 patients with TC. Our findings provide valuable insights into (1) somatic driver-derived neoantigens; (2) subtypes of TC with a high likelihood of response to ICIs and high production of neoantigens; and (3) recurrent gene fusions that give rise to alternative TSAs. Collectively, these findings shed light on our understanding of tumorigenesis and increase the therapeutic options in TC.

## 2 Materials and Methods

### 2.1 Patients and Clinical Data

Twenty-two subjects with TC were recruited for this study and received surgical resection at Shanghai Chest Hospital, Shanghai Jiao Tong University, China. Clinical information regarding TC patient history, along with relevant therapeutic information, was obtained from medical records and pathological reports. The study protocols were reviewed and approved by the medical ethics committee of the hospital. All eligible patients provided informed written consent prior to any study procedure or sample processing. All samples were enrolled in accordance with the WHO classification of TC. High-quality WES data was retrieved at an average depth of 100X from 19 TC patients with tumor and adjacent normal fresh-frozen samples, 8 of whom also had sufficient quality available for RNA sequencing (RNA-seq). Tumor-only RNA-seq datasets were collected from an additional 3 TC patients. These 22 TC patients were diagnosed between 39 and 80 years (median 60 years) of age; most were male (68%), and many were at stage III (Masaoka Koga stage: 52%; TNM stage: 41%). Detailed clinical information is summarized in **Table 1**. Details of pipelines used for downstream analyses are given in **Supplementary Methods** and **Supplementary Figures S1–4**, **S6–12**.

**Table 1:** Patient and sample characteristics.

Characteristics	Patient (n=22)*
Age, years (n=22)	
Median (interquartile range)	60 (53.25-72.00)
Distribution	
≤40	1 (5)
41–50	4 (18)
51–60	7 (32)
61–70	4 (18)
>70	6 (27)
Gender (n=22)	
Male	15 (68)
Female	7 (32)
Masaoka Koga stage (n=21)	
I	0 (0)
II	7 (34)
III	11 (52)
IV	3 (14)
TNM stage (n=22)	
I	6 (27)
II	1 (9)
III	9 (41)
IV	5 (23)

*All values are presented as n unless otherwise indicated.

### 2.2 Large-Scale Genomic Events Analysis

Three large-scale genomic events were evaluated across tumor samples and each is defined below in detail (**Supplementary Figure S1**).

#### 2.2.1 Whole-Genome Doubling (WGD)

Patients were considered to have undergone WGD if greater than 50% of their autosomal genome had a major copy number derived from FACETS (16) greater than or equal to two (17).

#### 2.2.2 Chromosomal Instability (CIN)

CIN is a broad concept that encompasses a wide range of chromosome-level abnormalities. The CIN burden is defined as the proportion of the genome in length affected by CNAs (18) and is given by:


$$\text{CIN}=\;\frac{\sum_{\text{i}=1}^{\text{n}}{\text{u}}_{i}}{\text{L}}$$

, where *L* is the total length of the autosome and *u_i_* represents the altered length in CNA *i*.

#### 2.2.3 Activated Telomere Maintenance Mechanism

To characterize the telomere maintenance mechanism, TelomereHunter (v1.1.0) (19) was run to quantify the content of 10 telomeric repeat types on whole-genome sequenced samples (n=4). The log_2_ ratio of telomere content was further summarized between tumor and normal samples.

### 2.3 Microsatellite Instability Identification

MSIsensor (v0.5) was implemented to distinguish tumors with MSI from stable samples in paired tumor-normal sequencing data (**Supplementary Figure S2**). The somatic status of relevant replication slippage variants at microsatellite regions was reported (20). Distributions of homopolymer regions with different lengths of oligonucleotides were aggregated in tumor and normal samples separately, and were compared by χ^2^ goodness-of-fit tests. The MSIsensor score was defined as the percentage of unstable sites. Patients with an MSI score ≥10% were regarded as MSI-high, while MSI scores of greater 3.5% and below 10% were classified as MSI-low; all others were considered microsatellite stable (21).

### 2.4 Prediction of Major Histocompatibility Complex-Binding Short Peptide Variants

As shown in **Supplementary Figure S3**, the Optitype algorithm (v1.3.2) was used to determine the 4-digit patient-specific human leukocyte antigen (HLA) class I typing from WES data (22). Prediction of MHC class I neoantigens for all possible 8- to 11-mer peptides spanning the somatic variant of interest was accomplished using NetMHC (v4.0) and NetMHCpan (v4.0) algorithms supported by pVACseq (v1.5.5) (23–25). Any mutant peptide that arose from the candidate variant with predicted binding affinity below 500 nM was defined as a neoantigen. The qualifying neoantigens were then annotated with a stability prediction and a cleavage position using pVACseq and assigned a relative rank to prioritize neoantigens.

### 2.5 Prediction of Major Histocompatibility Complex-Binding Neoantigens Arising From Gene Fusion

To identify neoantigens from gene fusions (**Supplementary Figure S4**), novel protein sequences resulting from gene fusion were initially annotated using AGFusion (v1.25) to predict functional effect and then used as input for pVACfuse (v1.5.5) (26). Neoepitopes ranging from 8 to 11 amino acids in length were constructed for fusion neoantigen candidates. Next, for each patient, 4-digit HLA class I typing from WES data was predicted using Optitype.

Binding strength between neoepitopes and the MHC was predicted using NetMHC and NetMHCpan algorithms within pVACfuse. Neoepitopes with binding affinity below 500 nM were reported as neoantigens. The qualifying neoantigens were then annotated with a cleavage position and underwent stability prediction by pVACfuse, with a ranking assessment for prioritizing neoantigens.

## 3 Results

### 3.1 Genomic Aneuploidy and Telomere Maintenance in TC

**Figure 1:**
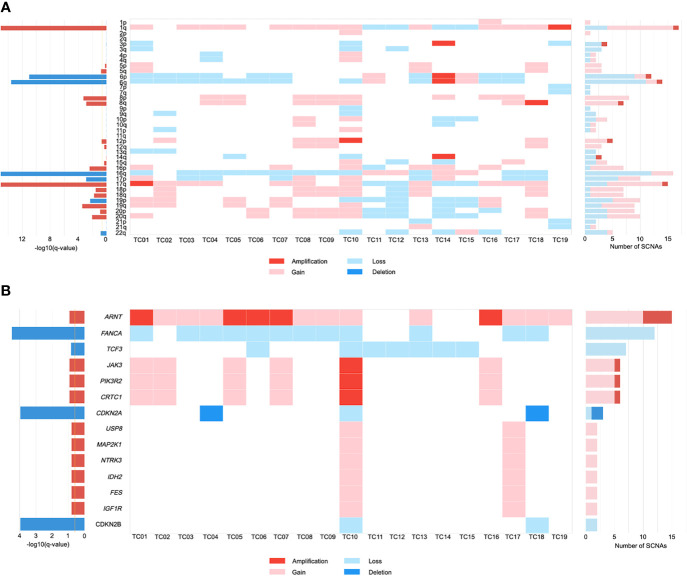
The landscape of somatic copy number alterations (SCNAs) in thymic carcinomas. **(A)** Heat map of SCNAs at arm level (row) for each patient (column). Color is coded by SCNA type. The bar chart to the left depicts the statistical significance [-log10(q-value)] of each arm. The bar chart to the right depicts the number of total SCNAs. **(B)** Heat map of SCNAs at gene level (row) for each patient (column). .

Somatic copy number alteration (SCNA) analysis revealed that five recurrently amplified and deleted regions were found more frequently at the chromosome arm level in TC patients. Overall, arm-level gains in chromosome 1q were present in a large majority (68%) of patients (**Figure 1A**). There were 12 out of 19 samples harboring a loss of chromosome 16q, which is consistent with previous observations (27, 28). Similarly, frequently recurrent arm-level losses involved chromosome 6 (6p, 47%; 6q, 58%) in our cohort. Notably, while looking for the novel SCNAs, we detected an enrichment in chromosome 17q gains (58%), which has not been previously reported in TC.

We further investigated the SCNAs that occurred within the amplified and deleted peaks containing known driver genes. These included significant focal gains/amplifications of the *ARNT* locus at 1q21.3 that occurred predominantly in our cohort (79%; **Figure 1B**). *JAK3*, *PIK3R2*, and *CRTC1* were recurrently amplified/gained at 19p13.11 in 32% of tumors. Focal gains that were identified at 15q11.2 in two patients (11%) included most of the putative driver genes, encompassing *USP8*, *MAP2K1*, *NTRK3*, *IDH2*, *FES*, and *IGF1R*. Similarly, evaluation of focal SCNA at 16q23.2 revealed an increased frequency of *FANCA* loss (63%). Inactivation of *CDKN2A* and *CDKN2B* were present at 9p21.3 in 16% and 11% of TCs, respectively. Focal SCNAs that were recurrent in 19p13.3 included deletions in *TCF3*. Based on these SCNA profiles, gene ontology (GO) term analysis suggested that TC progression might benefit from the dysfunction of protein tyrosine kinase activity and regulation of cell proliferation.

Furthermore, the CIN burden was analyzed by determining the proportion of the genome in length affected by SCNAs. Our results revealed a low CIN burden in our cohort (median: 0.25, IQR=0.19-0.32; **Supplementary Table S1**). Clinically, large-scale genomic events such as WGD are frequently associated with worse survival in cancer progression (29). One TC patient exhibited WGD, along with a greater degree of CIN (0.96; **Supplementary Table S1**). Notably, the burden of arm-level SCNA was enriched in the WGD-positive patient (TC10; **Supplementary Table S1**), who harbored all of the gene-level driver SCNAs (**Figure 1B**).

As an exploratory and speculative analysis, 4 paired whole-genome sequencing data were further analyzed by using TelomereHunter (19) to investigate the telomere maintenance mechanism in TC. Overall, the tumor samples had similar telomere content to the matched controls, with a median telomere content tumor/control log2 ratio of 0.04 (**Supplementary Figure S5**), and did not show a trend toward employing the alternative lengthening of telomeres pathway. Conversely, a significantly increased *TERT* expression was observed in tumor samples, indicating they relied on reactivating canonical telomere maintenance.

### 3.2 Correlation of Mutational Landscape and Burden With Immunogenicity in TC Patients

**Figure 2:**
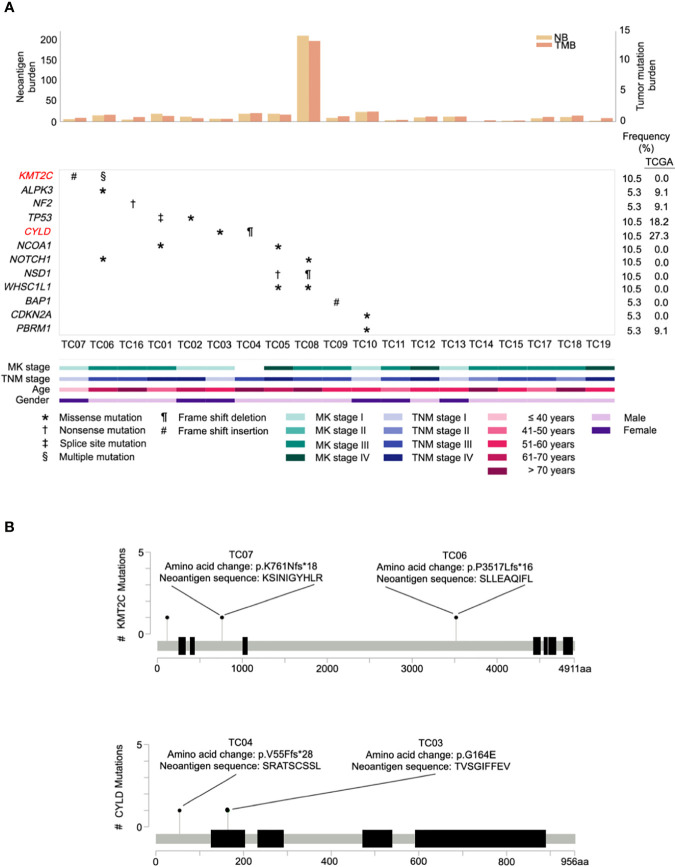
Repertoire of oncogenic alterations with neoantigens in thymic carcinoma patients (n = 19). **(A)** CoMut plot for each patient (column). Top: left y-axis for neoantigen burden, total number of predicted neoantigens; right y-axis for tumor mutational burden, i.e., mutations/covered bases. Middle: Row represents significantly mutated genes, curated driver genes of thymic carcinoma, and frequency of occurrence in our cohort and The Cancer Genome Atlas (TCGA) cohort. Significantly mutated genes are those exhibiting a significantly higher mutation rate than expected of the background mutation rate in the cancer cohort (30). Symbols indicate the type of somatic mutation. Bottom: Clinical characteristics of thymic carcinoma patients. MK stage, Masaoka Koga stage. **(B)** Lollipop plots of mutations for genes *KMT2C* and *CYLD* with predicted neoantigens. The heights indicate the number of mutations in patients. The amino acid change and its neoantigen sequence are annotated above the circles.

A survey of our data did not reveal any difference (excluding the hypermutated cases, *P*= 0.60) in median TMB between The Cancer Genome Atlas (TCGA) (0.83 mutations per megabase (mut/Mb); IQR=0.67-1.22) and our cohort (0.79 mut/Mb; IQR=0.55-0.95). Notably, similar to a previous observation (28), one TC patient (TC08) displayed a remarkably high TMB (13.15 mut/Mb; **Figure 2A**, top) in our cohort. As increased TMB is a key indicator of microsatellite instability (MSI) caused by mismatch repair defects (31), a scoring algorithm [MSIsensor (20)] was then applied to evaluate the MSI status for each patient. As expected, only the hypermutated sample was classified as MSI-high using a previously validated cut-off score (>10) (21).

To identify the significantly mutated genes (SMGs) in our cohort, after filtering one hypermutated case (TC08), we identified a gene, *KMT2C*, which has not been reported as a SMG in previous TC studies. *KMT2C* was altered in two samples that primarily harbored frameshift indels possessing high functional impact (**Figure 2A**, middle). A comparison of the mutation rate in *KMT2C* to TCGA dataset revealed prevalence only in our cohort (**Figure 2A**, right). Additional analyses of SMGs nominated 2 well-known TC driver genes, *TP53* and *CYLD*, under positive selection, and identified 2 additional SMGs, namely *NF2* and a novel SMG, *ALPK3*. The frequency of these 4 additional identified SMGs was then compared with the TCGA cohort. Genes *NF2* and *ALPK3* displayed a similar mutation rate in both cohorts, whereas *TP53* and *CYLD* were less frequently affected in our cohort (**Figure 2A**).

Next, to explore the landscape of potential cancer genes in our cohort, we manually curated a list of 12 frequently recurrent genes reported in two previous TC studies (28, 32) and also evaluated genes mined from the Cancer Gene Census (33). A total of 10 cancer genes were identified as drivers in our cohort (**Figure 2A**, middle), including *KMT2C*, *TP53*, *CYLD*, *NCOA1*, *NOTCH1*, *NSD1*, *WHSC1L1*, *BAP1*, *CDKN2A*, and *PBRM1*. Among these, seven genes were recurrently mutated in more than one case whereas three curated genes (*BAP1*, *CDKN2A*, and *PBRM1*) were sporadically altered in affected patients. Regarding our identified cancer genes, we found that, compared to TCGA cases, most genes were only identified in our cohort of Chinese patients (**Figure 2A**, right), implying that there is apparent genomic divergence across races.

Accumulating evidence suggests that active immunosurveillance could be therapeutically strengthened by immunotherapy (34). We subsequently investigated the potential neoantigens in our cohort. Putative neoantigens were computationally predicted based on somatic SNVs and indels. A median of 10 neoantigens was predicted across patients. Among those, two associated genes were recurrently identified, including an SMG, *KMT2C*, and a potential driver gene, *CYLD* (**Figure 2B**). Beyond mutations in *KMT2C* and *CYLD*, only frameshift deletion in *MOK* gave rise to four neoantigen products found in a single patient. As expected, the neoantigen burden was significantly correlated with elevated TMB (correlation coefficient 0.998, *P*<0.001), and the MSI-high case harbored exceptionally higher neoantigen burden (n=209) than other samples (**Figure 2A**, top; **Supplementary Table S2**), especially with increased frameshift indel-derived peptides from the higher frameshift indel burden.

### 3.3 Earlier Tumorigenesis From Normal Tissues, Gene Fusions for Immunogenicity, and Immune Cell Infiltration Across Paired Tissues

**Figure 3:**
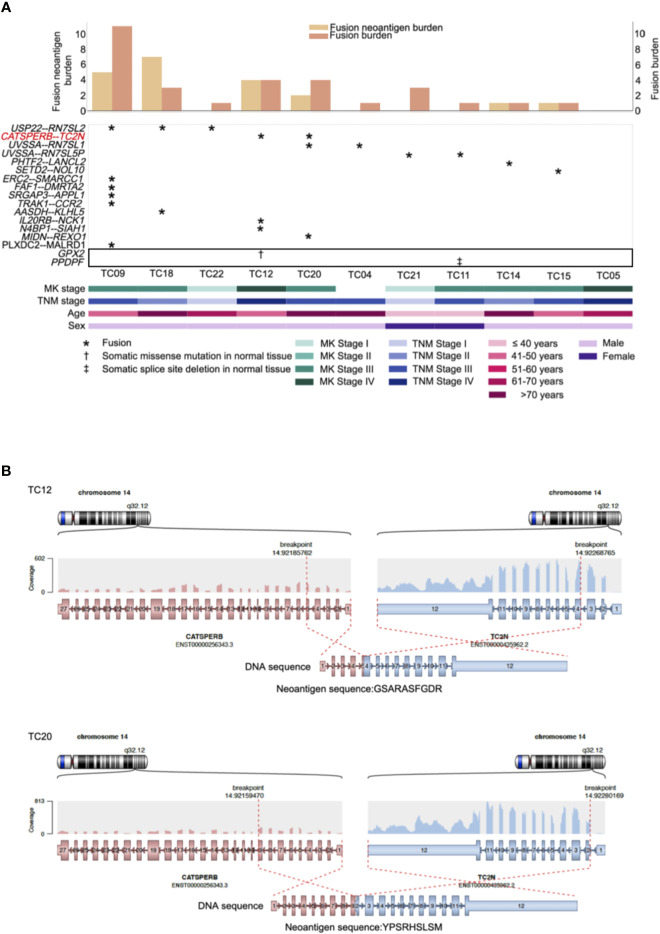
Repertoire of genes with neoantigens and somatic alterations in adjacent normal tissues of thymic carcinoma patients (n = 11). **(A)** CoMut plot for each patient (column). Top: left y-axis for fusion neoantigen burden, i.e., total number of predicted neoantigens; right y-axis for fusion burden, i.e., total number of fusions. Middle: Row represents predicted fusions and somatic alterations in the adjacent normal tissues (indicated in the box) of thymic carcinoma patients. Symbols indicate the types of alterations. Bottom: Clinical characteristics of thymic carcinoma patients. MK stage: Masaoka Koga stage. **(B)** The predicted *CATSPERB-TC2N* fusion-derived neoantigens in patients TC12 and TC20. Each plot depicts the fusion partners, their orientation, and the retained exons, with corresponding neoantigen sequences annotated below the fusion transcripts.

Recurrent gene fusions were further characterized to portray the full mutational portrait for 11 patients with RNA-seq data. Our results showed that 10 out of 11 patients (91%) had potential gene fusions. Given a median of two gene fusions present in each affected sample, these events were frequently present in patients with a low burden of recurrent driver mutations. Frequencies of gene fusions predicted in each patient are illustrated in **Figure 3A** (middle). In our cohort, the most recurrent fusion was *USP22*-*RN7SL2* (27%), in which the *USP22* generally had longer retained exons rearranged with exon 1 of *RN7SL2*. The other top recurrent fusion, *CATSPERB*-*TC2N*, was predicted to translate a novel fusion protein (**Figure 3B**).

**Figure 4:**
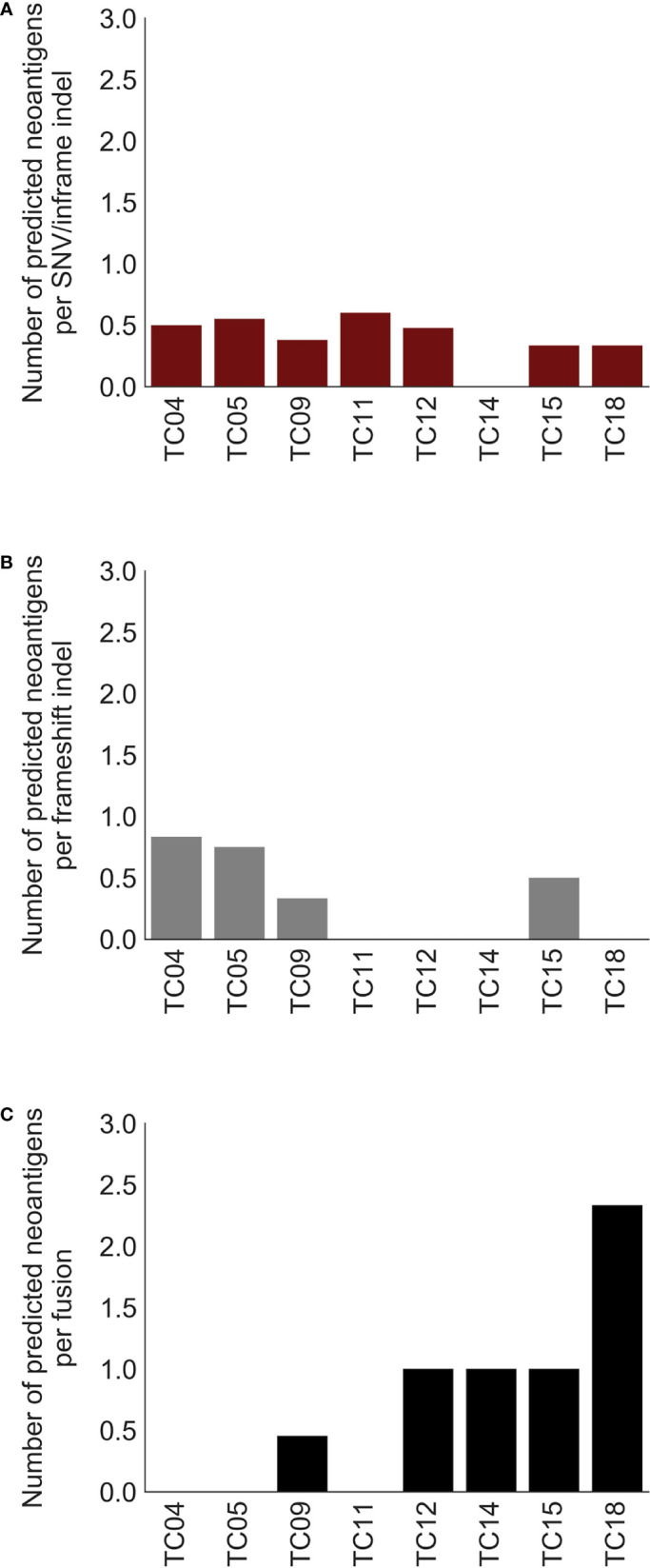
Comparison of predicted neoantigen candidates produced by three types of alterations. Bar charts of the number of predicted neoantigens per **(A)** SNV/inframe indel, **(B)** frameshift indel, and **(C)** fusion. X-axis: thymic carcinoma patients with both paired DNA and RNA data.

Previous evidence suggested that alternative TSAs derived from gene fusions may have better MHC binding capacity than SNV-derived neoantigens and may thus serve more effectively as therapeutic targets for vaccines (8). Subsequently, neoantigens produced by gene fusions were further investigated in our cohort. Our analysis showed that the fusion products were potentially immunogenic in 5 out of 10 affected patients (50%; **Figure 3A**, top), especially for recurrent fusion *CATSPERB*-*TC2N* (**Figure 3B**). Moreover, a median of 3 neoantigens was predicted across affected patients. Among this set, *AASDH*-*KLHL5* had the highest number of predicted neoantigen candidates, followed by *N4BP1*-*SIAH1* (7 and 2, respectively). Notably, there were 0.60 predicted potential immunogenic epitopes per fusion, compared to 0.47 per SNV/inframe indel and 0.53 per frameshift indel (excluded hypermutated cases), with higher values in patients with low non-fusion-derived neoantigen burden (**Figure 4**).

To explore the differences in cellular characterization of immune infiltrates across the 8 paired tumor and normal tissues, the CIBERSORTx algorithm (35) was employed to estimate the composition of 22 immune cell types for each sample. Compared to the matched normal tissues, macrophages M_0_, macrophages M_1_, and monocytes were significantly increased in tumor tissues (two-tailed, paired Mann-Whitney U test; *P*= 0.036, 0.008, and 0.023, respectively; **Supplementary Figure S6**). A higher proportion of activated dendritic cells were also found in tumor tissue (two-tailed, paired Mann-Whitney U test; *P* = 0.022). In contrast, the naive B cells, resting natural killer (NK) cells, and naïve CD4+ T cells were more abundant in normal tissues (two-tailed, paired Mann-Whitney U test; *P* = 0.039, 0.039, and 0.036, respectively). Taken together, these results showed the difference in immune function across tumor and matched normal tissues based on the differential composition of tumor-infiltrating immune cells.

As investigating somatic mutations in adjacent normal tissues or precancerous lesions may provide valuable insight into the initiation and progression of tumors, further analysis using RNA-MuTect (36) and Cancer Transcriptome Analysis Toolkit (CTAT)-Mutations pipelines revealed early macroscopic somatic clonal expansions. The *GPX2* mutation, which deleteriously alters protein function according to SIFT (37) and PolyPhen2 (38) predictions, was identified as a putative driver in normal tissues, as well as a splice site deletion in *PPDPF* (**Figure 3A**, middle). Overall, these alterations might play an important role on earlier tumorigenesis.

### 3.4 Actionability in TC

**Figure 5:**
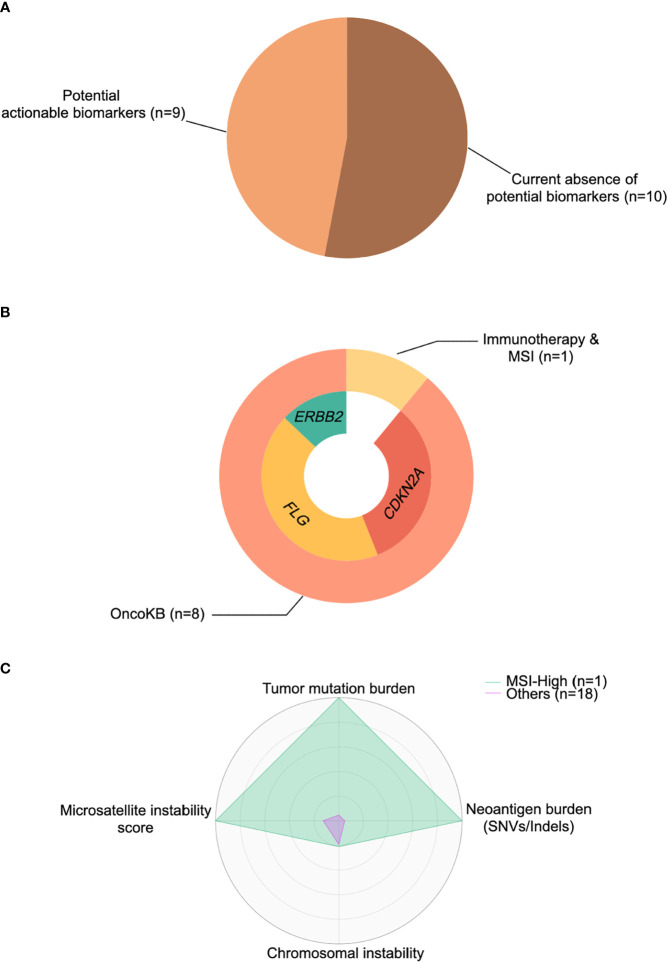
Cancer-immune interactions for actionable biomarkers in thymic carcinoma. **(A)** Percentage of patients with actionable biomarkers for treatment. **(B)** Percentage of patients with microsatellite instability (MSI)-high status and actionable biomarkers (*ERBB2*, *FLG*, *CDKN2A*) stratified by immunotherapy based on OncoKB database (39). **(C)** The representative radar plot of cancer-immune interactions to identify the thymic cancer patient amenable to immunotherapy. SNV, single nucleotide variant.

To investigate whether potentially actionable agents could mature into valuable predictive biomarkers and provide additional therapeutic choices for TC patients, we specifically focused on the aforementioned identified drivers and molecular features. These included: (1) hypermutated or/and MSI-high phenotype for immunotherapy; and (2) specific alterations with various levels of evidence cataloged in the OncoKB database (39). In accordance with these principles, we then calculated the percentage of TC patients with these events. Overall, 47% of tumors (n=9) had at least one actionable agent for currently available FDA-approved drugs or inclusion into future clinical trials for TC (**Figure 5A**).

OncoKB actionable alterations recapitulated in our cohort revealed 3 potential targets (**Figure 5B**), including *CDKN2A*, *FLG*, and *ERBB2*. Two patients harbored a *CDKN2A* mutation/deletion, which had compelling biological evidence (Level 4) with a predicted response to drugs in all tumor types. Moreover, four patients had an *FLG* amplification that might be treated by drugs registered for lung squamous cell carcinoma rather than TC, with compelling clinical evidence (Level 3A in OncoKB). Similarly, a single patient with *ERBB2* amplification raised the possibility that anti-HER2 therapies (Levels 1 and 2 in OncoKB) might be exploited in clinical trials for TC. Finally, as mentioned previously, one tumor was identified as hypermutated (TMB >10 mut/Mb) and MSI-high, with a higher SNV/indel-derived neoantigen burden than other samples (**Figure 5C**), and this tumor might be responsive/sensitive to pembrolizumab (which is approved for all solid tumors) (40).

## 4 Discussion

Uncovering the integrated genomic landscape of TC is critical for clinicians to understand the potential molecular mechanisms and to provide optimal treatment options. Our genomic analysis explored key attributes of TC such as the population-specific SMGs and driver gene-derived neoantigens. To comprehensively profile the whole portrait of TC, transcriptome-based changes were also leveraged for yielding insights, including the complementary role of recurrent gene fusion with the higher number of predicted neoantigens. In addition to providing fundamental research insights, our findings further identified actionable biomarkers for increasing therapeutic opportunities in TC.

The emergence of oncogenic mutation-derived neoantigens from the frameshift indels in *KMT2C* and *CYLD* raises the possibility of using driver genes in a vaccination strategy for TC, despite the fact that 92% of neoantigens are generated by passenger genes (13, 41). Although driver mutations have a relatively low likelihood of being presented by MHC-I (42) and MHC-II (43), which hinders the success of immunotherapy, frameshift indel-derived neoantigens have been hypothesized to elicit more robust immune responses in previous studies (15). The mutant peptides of frameshift indels encoded by novel open reading frames (neoORFs), which show lower similarity to the native genome, would contribute to immunogenicity (8, 11, 44). A recent study, which investigated the use of neoantigen vaccines in melanoma patients, prioritized epitopes derived from neoORFs in their pipeline (45). Furthermore, driver genes are required in order for tumor cells to tolerate chromosomal losses caused by CIN, while passenger gene-derived neoantigens may be lost without fitness impact to the cancer subclone (9, 44). However, not all neoantigens provide promising efficacy for patients (46). Like the tumor neoantigen selection alliance (TESLA) which established standards and functional validation methods to develop efficacious and safe personalized tumor vaccines, our identified neoantigens also required experimental validation to examine the immunogenicity for each patient. Collectively, it is worth considering the neoantigens from the frameshift indels of *KMT2C* and *CYLD* for further verification, which would narrow down the list of potential neoantigens for clinical trials in TC.

Although pembrolizumab showed encouraging clinical benefits in advanced TETs, with a response rate of 19% and 23% (6, 7), it needs to be identified who would benefit from ICIs without having to take the unnecessary risk of immune-related adverse effects. For example, a previous study suggested that such *CYLD* alterations were exclusively found in responders to pembrolizumab (47). Our whole-exome sequencing results also showed that *CYLD* were recurrently mutated in our cohort, indicating that pembrolizumab may be a potential ICIs for patients with thymic carcinoma. Notably, an important feature of TC is that, combined our data with the TCGA TC cohort, 7% of TC samples were identified as MSI-high, which is a larger proportion than most cancer types (48), notwithstanding considerable variability among patients. However, MSI has not been used as an inclusion criterion or for baseline treatment stratification in trials in TC patients (5–7). A higher level of MSI accompanied by higher TMB is associated with better response to pembrolizumab (49). Moreover, the frameshift indels can provide a greater number of predicted neoantigens, which might increase the chances of their use in peptide vaccination for MSI-high TC tumors (15), compared to neoantigens from SNVs. Evidence in previous studies for greater frameshift indel burden being correlated to high CD8^+^ T cell infiltration, owing to the deficiency of DNA mismatch repair pathways (50–53), implied that MSI-high TC tumors might be susceptible to immunotherapy. Similarly, we noted that our MSI-high TC patient showed evidence of an exceptionally high TMB and frameshift burden, with the greatest neoantigen burden in our cohort. Taken together, due to the characterization of higher immune cell infiltration and cytolytic activity in MSI-high tumors, TC patients with this subtype might have a better prognosis and therapeutic response to pembrolizumab than other TC patients in future clinical trials.

Our findings demonstrated that neoantigens derived from gene fusions could generate more predicted peptides than those from SNVs and indels, as a potentially complementary source for the cancer vaccine therapy. Based on recent studies, these neoantigens, especially those derived from frameshift fusions encoding novel peptides, were more dissimilar to self-antigen than SNV- and indel-derived ones, potentially allowing for greater immunogenicity (8). In our study, by performing an exploratory and speculative analysis, the identified *AASDH*-*KLHL5* fusion could probably drive this observation, which had seven predicted neoantigen candidates. Although gene fusions had lower frequency than SNVs and indels, recurrent fusion neoantigens were identified, such as *CATSPERB*-*TC2N*, which might be a potential target of off-the-shelf therapies. Evidence in adenoid cystic carcinoma with low TMB and lower immune infiltration demonstrated that recurrent *MYB*-*NFIB* fusions also exhibit immunogenic peptides in approximately 60% of patients (14). ICIs might work best with neoantigens that turn “cold” tumors to “hotter” ones for better antitumor immunity^10^, and are probably suitable in TC patients with low TMB. Although previous studies suggested *TC2N* and its partner gene, *CATSPERB*, playing important roles for tumorigenesis (54), it remained uncertain for the immunogenicity of *CATSPERB*-*TC2N* fusion-derived neoantigen. Therefore, further experimental work is required to examine the use of these fusion neoantigen candidates in clinical trials with rigorous immunological validation. Moreover, the immune cellular composition estimates showed that macrophages M_0_, macrophages M_1_, monocytes, and activated dendritic cells were more abundant in tumor tissues. The resting NK cells and naïve CD4^+^ T cells were decreased in tumor tissues. It might suggest promoting tumor progression and related mechanisms in immunosuppression (55).

Additional findings from this study were the identification of a WGD-positive TC tumor and actionable biomarkers for therapies. As WGD is an early clonal event in tumor progression, neoantigens derived from pre-WGD alterations might increase the likelihood of T cell recognition compared to those occurring post-WGD, owing to the higher allelic fraction (44). Thus, to enhance the therapeutic efficiency in TC tumors with the WGD phenotype, clinicians could prioritize neoantigens for cancer vaccination based on the timing of WGD and somatic alterations. Moreover, further explorations and clinical trials could consider alterations in *CDKN2A*, *FLG*, and *ERBB2* to increase the targetability of the TC genome, in spite of only early levels of evidence.

Regarding the somatic mutations in adjacent normal tissues, we found that somatic mutations in *GPX2* and *PPDPF* initially occurred in normal tissues patients and accumulated in tumors. *GPX2* plays a role in protecting cells against oxidative damage (56), and *PPDPF* is a probable regulator of exocrine pancreas development (57). Abnormal expression of *GPX2* might facilitate tumorigenesis and result in poor progress in adrenocortical cancer, kidney papillary cell carcinoma, and uveal melanoma (56). Aberrant *PPDPF* expression was correlated with poor prognosis in hepatocellular carcinoma (58). Based on a previous study (36), we hypothesized that somatic mutations occurring in normal tissues would potentially represent the earliest stages preceding cancer initiation. Yet, future studies were still warranted to experimentally validate such potential somatic drivers in normal tissues for new insight into thymic tumorigenesis.

Overall, our analyses might have important implications for understanding the selection of neoantigens, as well as actionable biomarkers for additional therapeutic choices in TC. The comprehensive mutational landscape might also provide valuable insight into the biology of thymic carcinogenesis. Importantly, more TC samples are needed to validate our findings, so as to provide further clinical measures and verification to carefully confirm the immunogenicity. Although the molecular mechanisms of TC remain an understudied area, our study will help shape perspectives on its genomic characterization, which was of particular interest given the link between alternative TSAs and immunotherapy.

## Data Availability Statement

The original contributions presented in the study are publicly available. This data can be found here: https://www.ncbi.nlm.nih.gov/bioproject/PRJNA751999.

## Ethics Statement

The studies involving human participants were reviewed and approved by the medical ethics committee of Shanghai Chest Hospital, Shanghai Jiao Tong University, China. The patients/participants provided their written informed consent to participate in this study.

## Author Contributions

EC and L-CL designed the study. M-HT, L-HC, and Q-LS performed the experiments. Collection of specimens was coordinated by WF, Z-TG, LZ, TM, X-FZ, and NX. Clinical data was organized by WF, Q-LS, and T-PL. Data analyses were conducted by C-HW and L-CL. C-HW performed statistical analyses. WF, C-HW, Q-LS, L-CL, and EC wrote the manuscript. All authors contributed to the article and approved the submitted version.

## Funding

This work was supported by a grant from the Ministry of Science and Technology [MOST 109-2320-B-002-016-MY3], Strategic Priority Research Program of the Chinese Academy of Sciences (No.XDA12020101 to JD), National Natural Science Foundation of China (Nos.82073285 and 82072569), Interdisciplinary Program of Shanghai Jiao Tong University (No.ZH2018ZDA27), Shanghai Chest Hospital Project of Collaborative Innovation (YJXT20190104), Nurture Projects for Basic Research of Shanghai Chest Hospital (2018YNJCM06), and Shanghai JiaoTong University, College of Medicine Biobank Fund (YBKL2013009).

## Conflict of Interest

The authors declare that the research was conducted in the absence of any commercial or financial relationships that could be construed as a potential conflict of interest.

## Publisher’s Note

All claims expressed in this article are solely those of the authors and do not necessarily represent those of their affiliated organizations, or those of the publisher, the editors and the reviewers. Any product that may be evaluated in this article, or claim that may be made by its manufacturer, is not guaranteed or endorsed by the publisher.
