# Supplementary material for: Novel Tumor-Specific Antigens for Immunotherapy Identified From Multi-omics Profiling in Thymic Carcinomas
Source: Front Immunol. 2021 Nov 16;12:748820. doi: 10.3389/fimmu.2021.748820 (PMC8635231; doi:10.3389/fimmu.2021.748820)
Supplement: Supplementary file 1 [file DataSheet_1.docx]

Supplementary Material

# Supplementary Methods

## Whole-exome sequencing

For preparing the standard capture libraries, Agilent SureSelect XT Human All Exon Version 6 was used for genomic DNA samples. Libraries were quantified using the Agilent 2100 Bioanalyzer. All samples were sequenced on an Illumina Hiseq2500 instrument using the 150PE protocol. Low-quality sequences and adaptors from the raw Illumina sequence data were trimmed. The average sequencing depth of WES was more than 100 in both tumor and matched normal tissues.

## Short peptide variant and neoantigen annotation

In Fig. S3, somatic non-synonymous SNVs and indels were initially annotated using Ensembl-Variant Effect Predictor (VEP) with wild-type and downstream plugins(1). These plugins provided the wild-type transcript protein sequence in the annotation and computed the downstream effects of frameshift variants on the protein sequence. In order to be accepted as input by the following programs, somatic multiallelic variants were subsequently split using vt(2). Furthermore, since calculated protein sequences near the somatic variants of interest might be misled by the altered amino acids, proximal somatic and germline missense variants were then combined and phased using GATK CombineVariants and ReadBackedPhasing(3).

The qualifying neoantigens were annotated with a stability prediction and a cleavage position using the NetMHCStabPan(4) and NetChop(5) algorithms within pVACtools (pVACseq and pVACfuse) and assigned a relative rank to prioritize neoantigens.

## Allele-specific copy number alteration

The FACETS algorithm (v0.5.2) was used to call allele-specific copy number alterations (CNAs)(6) based on heterozygous single nucleotide polymorphism (SNP) sites, which were catalogued in the dbSNP and 1000 genomes (1000G) databases (Fig. S7)(7). The outputs of FACETS included allelic CN/CN state, purity/ploidy, clonality, and logR/logOR.

To discover the recurrent CNAs, we analyzed both arm-level and focal copy number calls based on GISTIC2 (v2.0.23)(8). Significantly recurrent CNAs were identified based on the median log_2_ ratio from FACETS with q-value <0.25. The significance of arm frequencies was controlled by using a linear model for the relationship between frequency of alteration and the number of genes.

Two estimators, GISTIC2 significance (q-value) and event frequency, were sequentially considered to determine the impact of focal CNAs. The gene-level amplification/deletion values produced by GISTIC2 were also used in this analysis, considering homozygous deletion, heterozygous loss, additional copy gain, and high-level amplification with clinical impact. To generate a list of recurrent gene-level CNAs, altered genes present in OncoKB(9), CGC(10), and Signaling pathway(11) were reported. All tools were run using the default parameters.

## Somatic variant calling and post-processing of sequencing data

The Bioinformatics pipelines were followed per the GDC data user guide (<https://docs.gdc.cancer.gov/Data/PDF/Data_UG.pdf>). Briefly, as shown in Fig. S8, fastq data of tumor and matched normal DNA samples were mapped to the human reference genome (hg19) using BWA-MEM (v0.7.15)(12), and sorted by chromosomal coordinate by the Picard module (v2.6.0; <https://broadinstitute.github.io/picard/>). The MarkDuplicates tool in Picard was used to mark PCR and optical duplicates.

The resulting BAM files were further processed following the Genome Analysis Toolkit (GATK) Best Practices (v3.7) to correct alignment and sequencing errors(3). First, indel realignment was implemented at intervals around the Mills and 1000G gold standard INDELs (Mills set)(13) to improve the mapping accuracy. Then an accurate confidence score for each base was calculated by recalibrating the base quality score using known variants in dbSNP138 and the Mills set(14). To refine variant calling near the boundary of target regions, 100 bp flanking the target regions were included for analysis.

Next, MuTect2 (v3.7.0) was used to conduct somatic SNV and indel calling in targeted exons from matched tumor-normal pairs(15), utilizing the Catalogue of Somatic Mutations in Cancer (COSMIC) and dbSNP138 as references(16). False-positive, systematic bias, and miscalled germline variants were excluded by constructing a panel-of-normals as a filter using MuTect2 on all the genomes of normal samples. Biased variants from cross-sample contamination were also discarded by MuTect2.

Identified variants were further filtered as follows: (1) removing initial variants within the non-targeted region using SelectVariants; (2) removing sequence context-dependent artifacts on the PCR template strand and bias on the forward/reverse strand using DKFZBiasFilter (v1.2.3; (<https://github.com/DKFZ-ODCF/DKFZBiasFilter>); (3) removing likely 8-oxoguanine error variants, caused by excessive oxidation during sequence library preparation, using D-ToxoG(17); and (4) removing variants with population allele counts ≤16 across at least one Exome Aggregation Consortium (ExAC) in the non-TCGA subpopulation (v0.3.1)(18). This filtering setting was the same as the TCGA MC3 project(19).

The DeTiN algorithm (v2.0) was utilized to rescue the discarded calls due to potential tumor-in-normal contamination(20). Somatic SNVs and indels were annotated, and a mutation annotation format (MAF) file was produced using Variant Effect Predictor (VEP v98.3)(1) and a vcf2maf.pl script (<https://github.com/mskcc/vcf2maf>).

To obtain cohort-specific genetic features, 11 cases with TC in the TCGA database were also compared, and all mutations were via post-filtering strategies based on the TCGA MC3 project. The clinical data were retrieved from cBioPortal for Cancer Genomics(19, 21).

## Strategy for discovering significantly mutated genes

Significantly mutated genes (SMGs) describe genes that exhibit a significantly higher mutation rate than expected of the background mutation rate in the cancer cohort(22). To discover SMGs, hypermutated cases (>500 SNVs and indels) were excluded to reduce the artifactual sensitivity due to high background mutation rates. A comprehensive suite of analytic tools (Fig. S9) across four selection-related features, including a recurrent-based tool (dNdScv), a clustering-based tool (oncodriveCLUST), a rule-based tool (20/20+), and a functional impact-based tool (oncodriveFML), were implemented to create a consensus list of SMGs. (1) dNdScv (v0.1.0), a maximum-likelihood method for calculating the normalized ratio of non-synonymous to synonymous mutations (dN/dS), quantified positive selection of missense, nonsense, and splice mutations at the level of individual genes, groups of genes, or the whole genome in cancer and somatic cell evolution(23). Genes with a q-value <0.1 were considered significantly mutated. (2) OncodriveCLUST (v1.0.0), a method used to identify oncogenes with a significant bias towards clustering mutations, identified genes with more nonsynonymous mutations than expected(24). Genes with a q-value <0.05 were considered significantly mutated. (3) OncodriveFML (v2.0.3) is a method to identify genes with an accumulation of mutations that also possess high predicted functional impact in various genomic regions(25). The CADD score was used to evaluate the functional impact of each mutation(26). Genes with a q-value <0.25 were considered significantly mutated. (4) 20/20+ (v1.1.3), a random forest-based and ratiometric method, classifies genes as oncogenes and tumor suppressor genes from somatic mutations based on the 20/20 rule(27, 28). Genes with a q-value <0.05 were considered significantly mutated. All the significance thresholds were based on a previous study(29), and all tools were run using default settings.

## Potential driver and actionable gene identification

To uncover the potential driver gene landscape, missense and loss-of-function (LOF) mutations were considered for analysis. LOF mutations were defined as those with clear functional impact, including frameshift, nonsense, start/stop codon changes, and splice site mutations. Genes were considered as potential drivers for further analysis if they were present in the repositories of the COSMIC Cancer Gene Census database or previous literature(30, 31). In addition, the known actionable genes were summarized based on the annotation of levels of evidence in the OncoKB database(9).

## RNA sequencing

For generation of cDNA sequencing libraries, the KAPA Stranded RNA-Seq Library Prep kit from Illumina was used for RNA samples. Quality control was employed for every step, and libraries were quantified using the Agilent 2100 Bioanalyzer. Prepared libraries were sequenced on an Illumina Hiseq4000 instrument using the 150PE protocol. Low-quality sequences and adaptors from the raw Illumina sequence data were trimmed. On average, 40 million paired-end reads were generated for tumor and matched normal tissues.

## Detection of gene fusions

The workflow for identifying fusion genes is shown in Fig. S10. mRNA sequencing reads of both normal and tumor samples were aligned to a human genome reference (hg19) by STAR (v2.7.2b) using the 2-pass model(32). A combination of three fusion detection tools was implemented to create a robust consensus list of fusions: (1) STAR-fusion (v1.8.0), a component of the Trinity Cancer Transcriptome Analysis Toolkit (CTAT), mapped STAR-aligned junctions and spanning reads to a reference annotation set(33). Resultant fusions were further filtered to remove artifacts by using FusionFilter, including fusion paralog, promiscuous fusion, red herring, and fusion expression filters. The predicted fusions were validated by the FusionInspector, which realigned reads and re-scored predictions. The fusion events, which generated novel fusion proteins, were also examined for impact on coding regions. The final predicted fusions were checked and refined by the Trinity-reconstructed fusion transcript(34). The STAR-Fusion pipeline leveraged a precompiled bundle of genomic resources (CTAT genome lib StarFv1.8) with default settings. (2) STAR-SEQR (<https://github.com/ExpressionAnalysis/STAR-SEQR>; v0.6.7), an algorithm designed for fusion-directed clinical diagnostics and immunotherapy, used the network graph approach and cross-homology score to disambiguate multiple mapping. (3) Arriba (<https://github.com/suhrig/arriba/>; v1.2.0), the winner of the DREAM SMC-RNA Challenge, was also employed. All tools were run using default parameters.

As STAR-Fusion showed a higher sensitivity for fusion detection in previous TCGA studies, we generated the starting set of fusion candidates by its output and integrated STAR-SEQR and Arriba output in the following filtering steps: (i) fusions detected in normal tissues were filtered; (ii) fusions involving irrational gene annotation of callers were filtered; (iii) fusions reported by only STAR-Fusion with >0.1 fusion fragments per million (FFPM) total reads were retained; and (iv) fusions reported by at least two callers were retained (no minimum FFPM was required).

## Immune cellular composition estimates

To characterize lymphocyte infiltration in TC, for each sample, HISAT2 (v2.0.3beta)(35) was used to map RNA-seq reads to the hg19 reference genome, and StringTie (v2.0)(36) was subsequently utilized to quantify the transcript abundances (Figs. S11). We also implemented the tximport (v1.20.0)(37) package to summarize transcript-level abundance estimates to the gene-level as the input for CIBERSORTx(38). The composition of 22 tumor-infiltrating immune cell types from 8 TC patients was further assessed using web-based CIBERSORTx (<https://cibersortx.stanford.edu/index.php>), which employed immune cell reference derived from healthy peripheral blood leukocytes as the signature matrix (LM22).

## Short variant discovery by RNA-seq

Macroscopic somatic clonal expansion in normal tissues potentially represents the earliest stage of tumorigenesis. Normal tissue-derived RNA samples and matched DNA samples were used to identify somatic variants by applying the RNA-MuTect pipeline (v1.0)(39) and the CTAT-Mutations pipeline (v2.3.0b; <https://github.com/NCIP/ctat-mutations>) (Figs. S12-S14).

As shown in Fig. S12, the RNA-MuTect pipeline was implemented in the following steps: (1) utilizing MuTect to STAR-align RNA-seq BAMs (cases) with matched DNA-seq samples (control) and retaining variants supported by at least 3 mutant reads; (2) filtering artifact variants by a DNA panel-of-normals; (3) retaining consensus mutations called in BAMs from two alignment tools (STAR and HISAT2(35)). To ensure that variants were not called from biases of different alignment tools, all reads aligned to the spanning position of candidate variants from STAR-aligned RNA and BWA-aligned matched DNA BAMs were extracted. These reads were further realigned using HISAT2 (v2.0.3beta) and recalled by MuTect by: (4) filtering artifact variants by an RNA panel-of-normals; (5) eliminating common variants found in the ExAC database with minor allele frequency more than 5%; (6) retaining variants supported by at least 3 nonduplicated reads; (7) filtering artifact variants caused by sequencing leakage errors; (8) filtering variants in non-coding regions, pseudogenes, or IgG genes; and (9) eliminating RNA editing variants listed in the RADAR and DARNED databases(40, 41).

The RNA somatic variants in normal tissues were also called using the CTAT-Mutations pipeline (Fig. S13). The RNA-seq short variant discovery workflow applied GATK4 Best Practices to STAR-aligned RNA-seq BAMs. The steps of filtering variants included (1) removing the likely RNA-editing events by leveraging the RediPortal and RADAR databases(40, 42), (2) excluding common variants found in dbSNP or the gnomAD database(43), (3) annotating known cancer mutations in the COSMIC database(16), and (4) prioritizing variants based on likely biological effect and relevance to cancer using CRAVAT(44).

Lastly, as shown in Fig. S14, combining the RNA-MuTect workflow and the CTAT-Mutation workflow, only variants identified by both workflows and present in tumor tissues (somatic variants from DNA-based workflow) were considered. Remaining missense variants, predicted as deleterious from SIFT and/or possibly/probably damaging from PolyPhen(45, 46), or loss-of-function variants were considered as the potential somatic events involved in the earliest stages of tumorigenesis.

## Statistics

Pearson correlation coefficients were calculated to correlate neoantigen burden and tumor mutation burden, along with its significance test (derived from the Student's t-distribution), using R version 3.4.3 (<https://www.r-project.org/>). The differences in the composition of 22 tumor-infiltrating immune cell types across 8 tumor and matched normal tissues were analyzed using a two-tailed, paired Mann-Whitney U test.

# Supplementary Figures


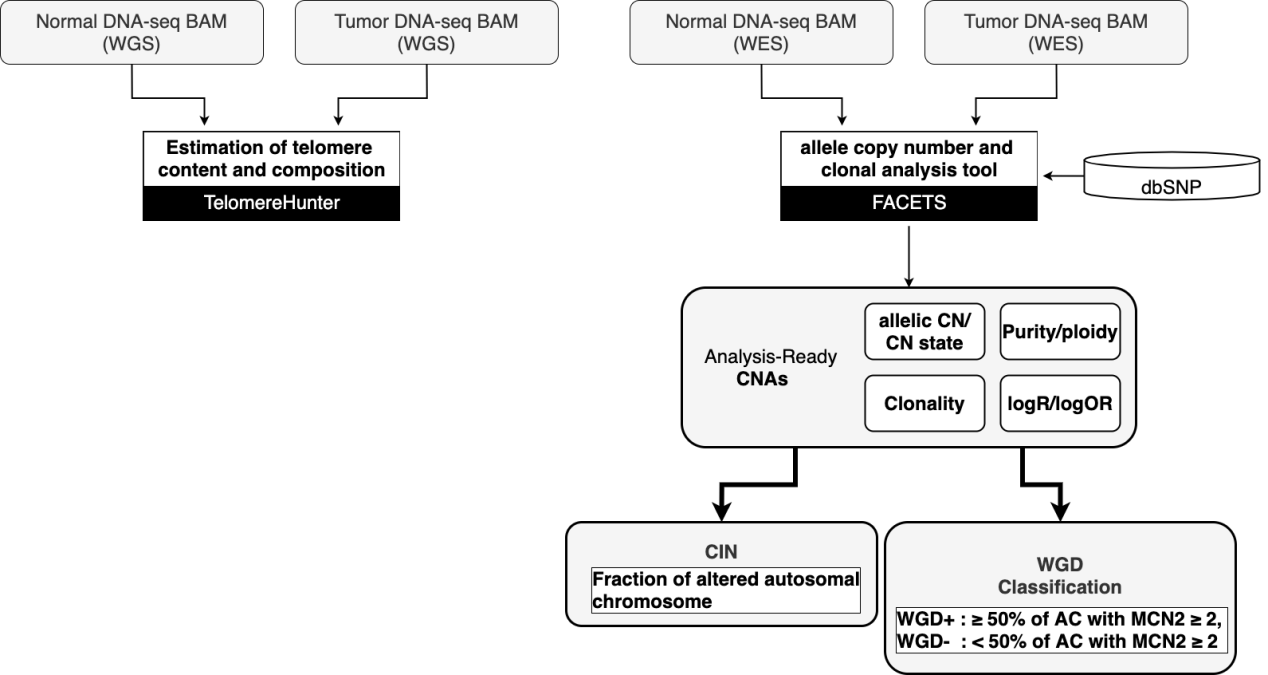


**Figure S1. Workflow for detecting large-scale genomic events in tumor tissues.** This workflow focused on detecting whole-genome doubling and chromosomal instability in tumor tissues. TelomereHunter: v1.1.0(47). AC: autosomal chromosome; BAM: binary alignment map file; CIN: chromosomal instability; CN: copy number; CNA: copy number alteration; MCN: major copy number; WES: whole-exome sequencing; WGD: whole-genome doubling; WGS: whole-genome sequencing.


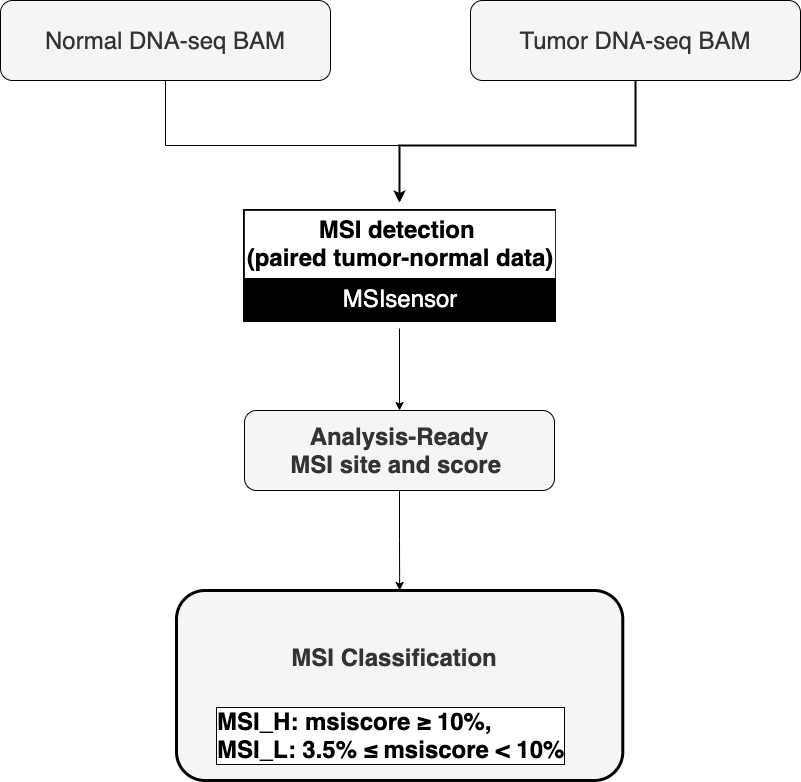


**Figure S2. Workflow for classifying microsatellite instability.** MSIsensor: v0.5(48). BAM: binary alignment map file; MSI: microsatellite instability; MSI-L: low microsatellite instability; MSI-H: high microsatellite instability.


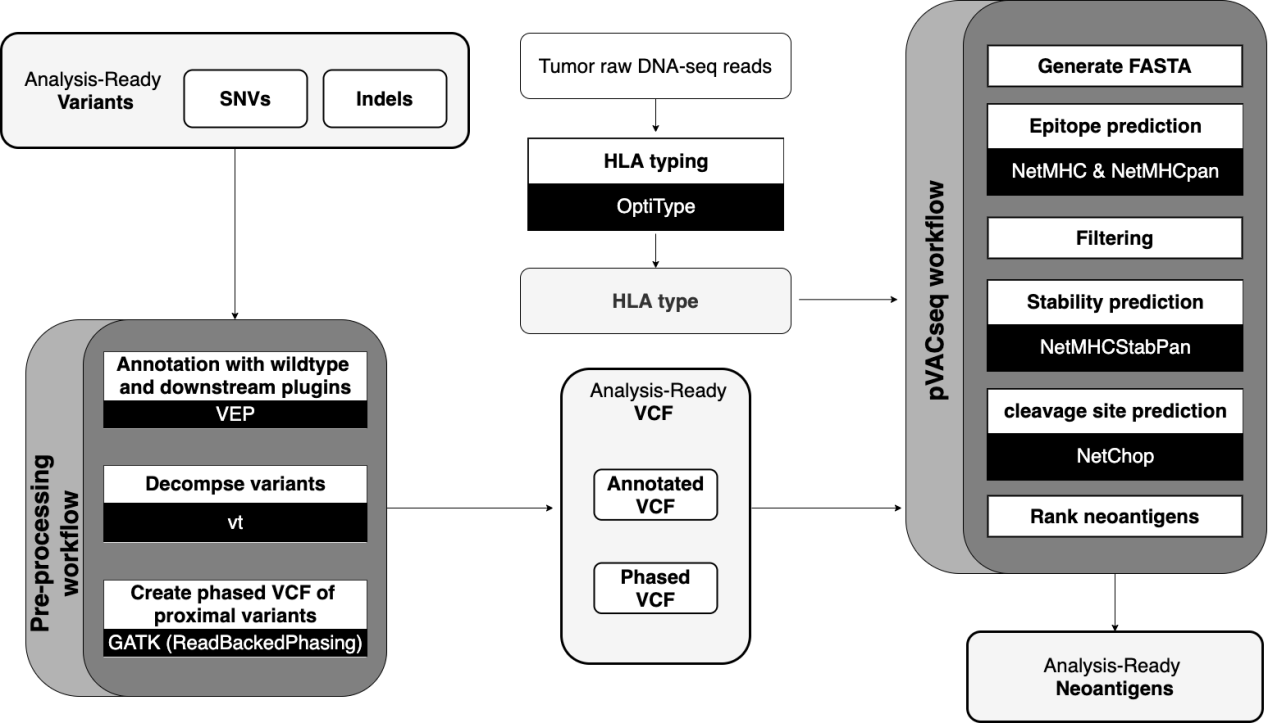


**Figure S3. Workflow for predicting neoantigens based on somatic short variants using the pVACseq pipeline.** GATK (ReadBackedPhasing): v3.7^3^; NetMHC: v4.0(49); NetMHCpan: v4.0(50); NetMHCStabPan: (4); NetChop: (5); OptiType: v1.3.2(51); pVACseq: v1.5.5(52) ; VEP: v98.3(1); vt(2). HLA: human leukocyte antigen; SNV: single nucleotide variant; VCF: variant call format.


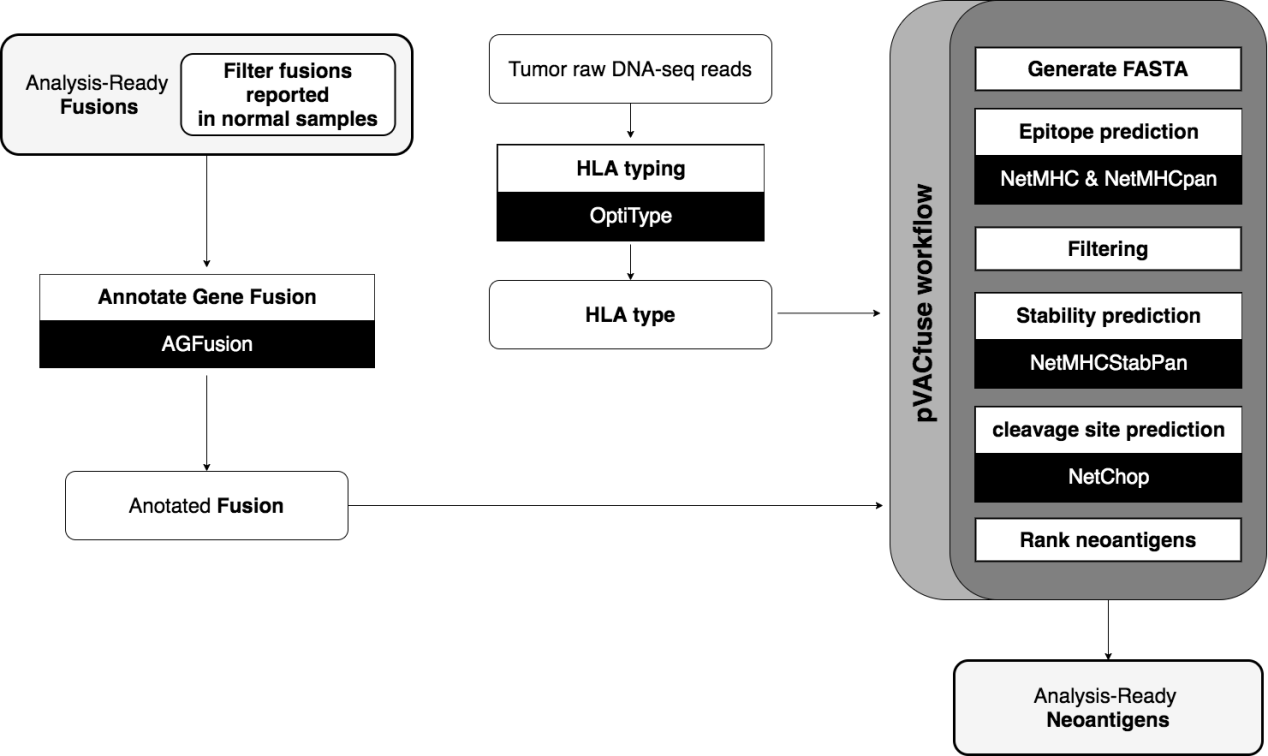


**Figure S4. Workflow for predicting MHC-binding neoantigens from gene fusions.** AGFusion: v1.25(53); NetMHC: v4.0(49); NetMHCpan: v4.0(50); NetMHCStabPan(4); NetChop(5) ; OptiType: v1.3.2(51); pVACfuse: v1.5.5(52). HLA: human leukocyte antigen.


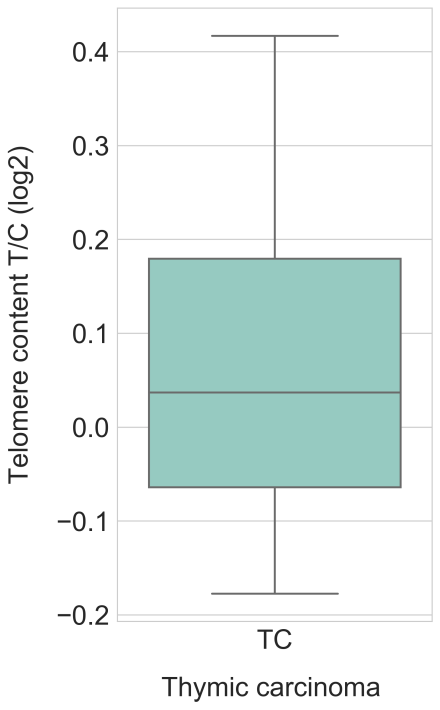


**Figure S5.** **Telomere content distribution in thymic carcinoma.** Log_2_ ratio of telomere content between tumor and control samples.


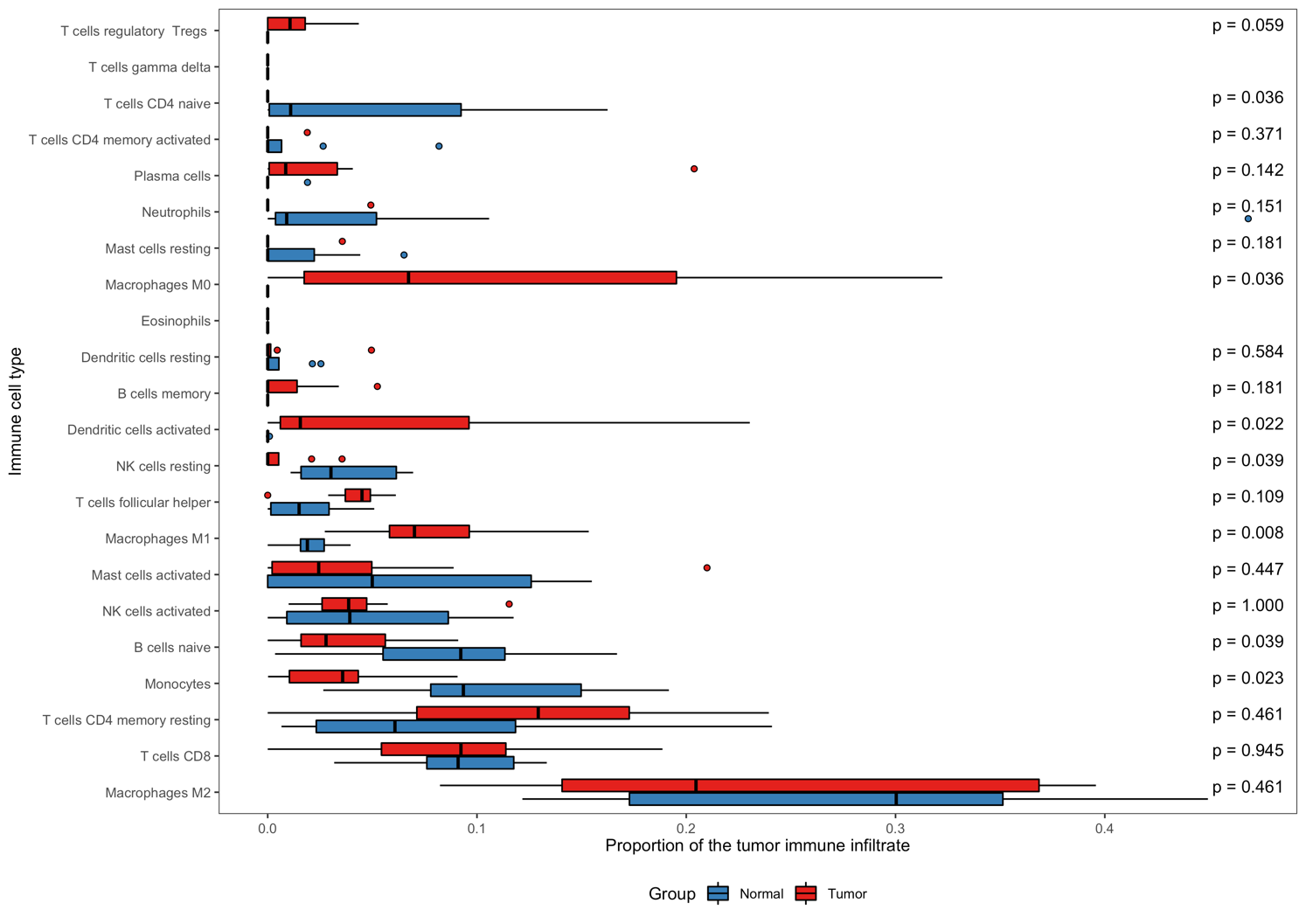


**Figure S6. Proportion of immune cellular composition across paired tumor and normal tissues**. The box plots indicate the composition of 22 tumor-infiltrating immune cell types across 8 tumor and matched normal tissues. Color is coded by tissue type. The two-tailed, paired Mann-Whitney U test was used to calculate the p-values for the comparison of medians.


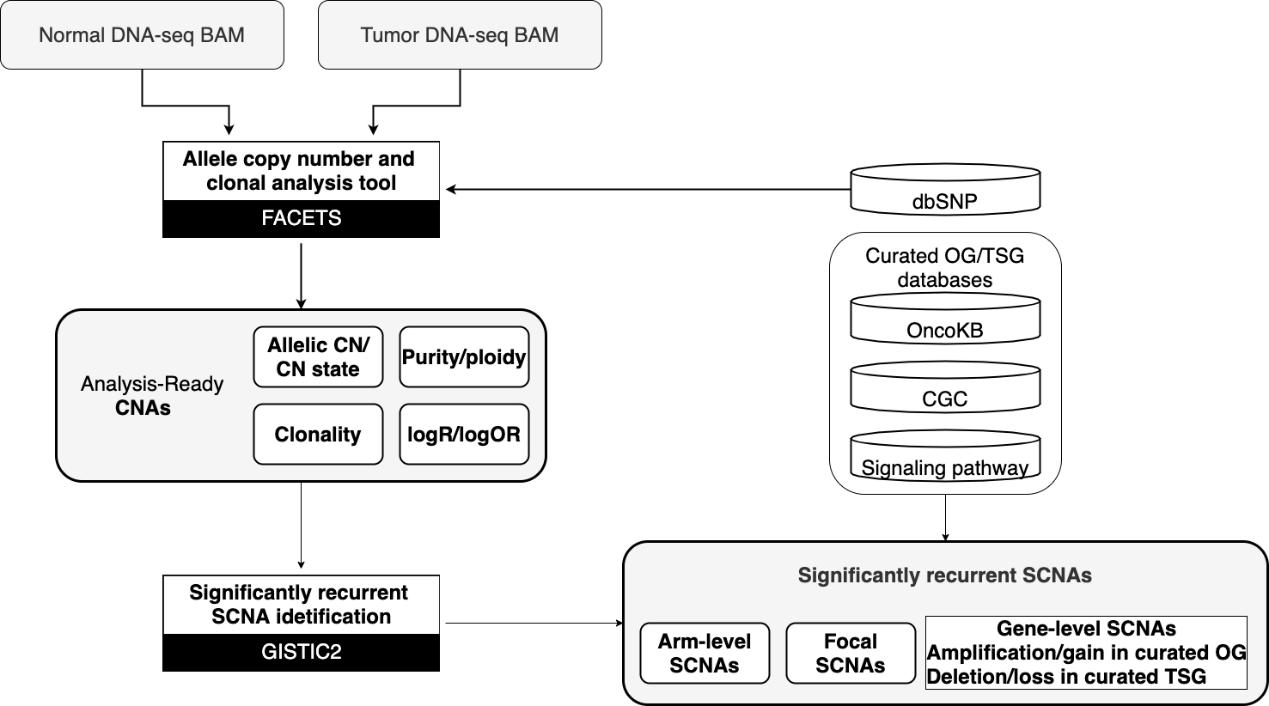


**Figure S7. Workflow for analyzing recurrent copy number alteration.** CGC(10); dbSNP: v151(14); FACETS: v0.5.14(6); GISTIC2(8); OncoKB(9); Signaling pathway(11). BAM: binary alignment map file; CN: copy number; CNA: copy number alteration; logR: total copy number log-ratio; logOR: allelic log-odds-ratio; OG: oncogene; SCNA: somatic copy number alteration; TSG: tumor suppressor gene.


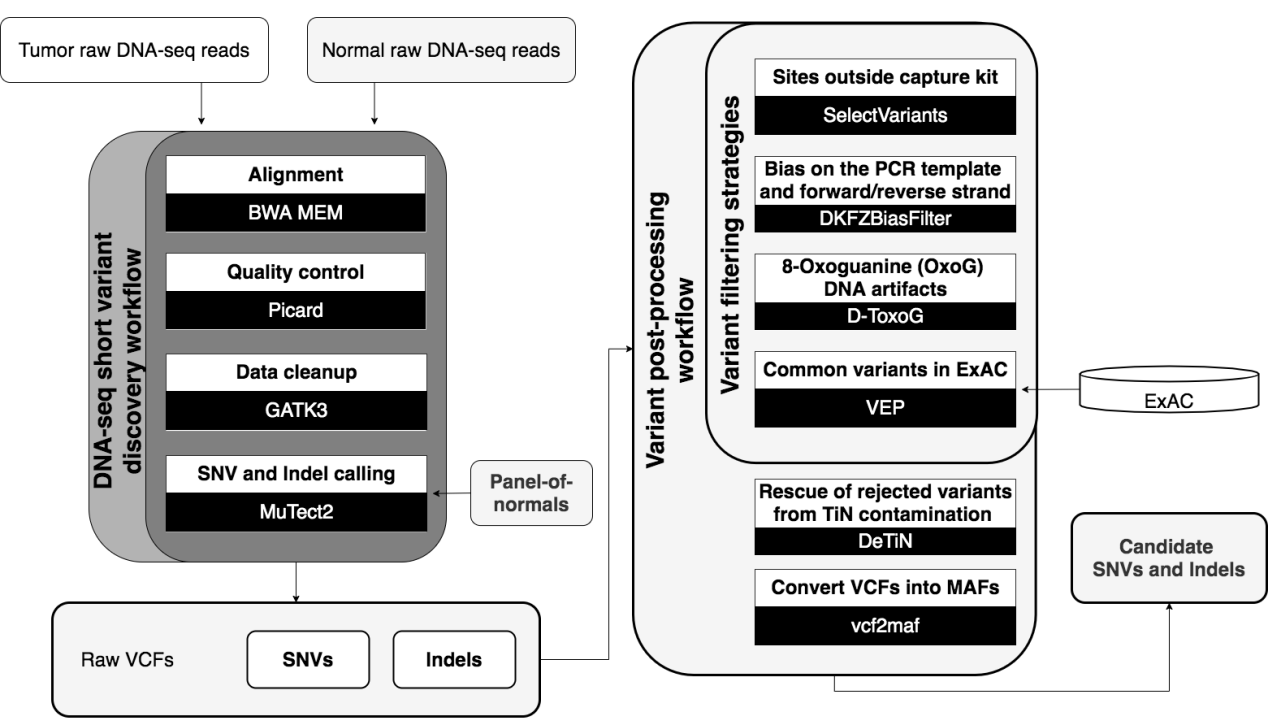


**Figure S8. Workflow for somatic variant calling and post-processing.** BWA-MEM: v0.7.15(12); D-ToxoG: (<https://software.broadinstitute.org/cancer/cga/dtoxog>)(17); DeTiN: v2.0(20); DKFZBiasFilter: v1.2.3 (<https://github.com/DKFZ-ODCF/DKFZBiasFilter>); ExAC: v0.3.1(18); GATK (SelectVariants; MuTect2): v3.7(3, 15); Picard: v2.6.0 (<https://broadinstitute.github.io/picard/>); VEP: v98.3(1); vcf2maf: v1.6.17 (<https://github.com/mskcc/vcf2maf>). MAF: mutation annotation format; SNVs: single nucleotide variant; TiN: tumor-in-normal; VCF: variant call format.


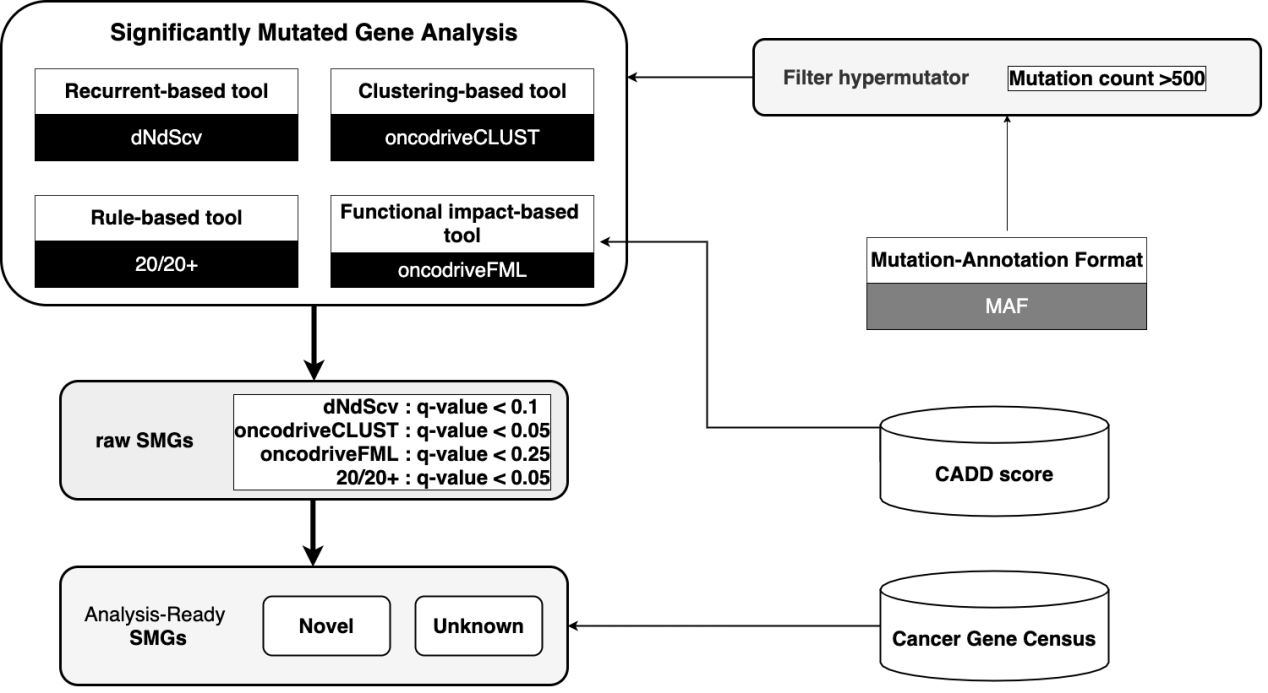


**Figure S9. Significantly mutated gene analysis.** 20/20+: v1.1.3^16,^; CADD score(26); Cancer Gene Census(10); dndScv: v0.1.0(23); OncodriveCLUST: v1.0.0(24); OncodriveFML: v2.0.3(25). MAF: mutation annotation format; SMG: significantly mutated gene.


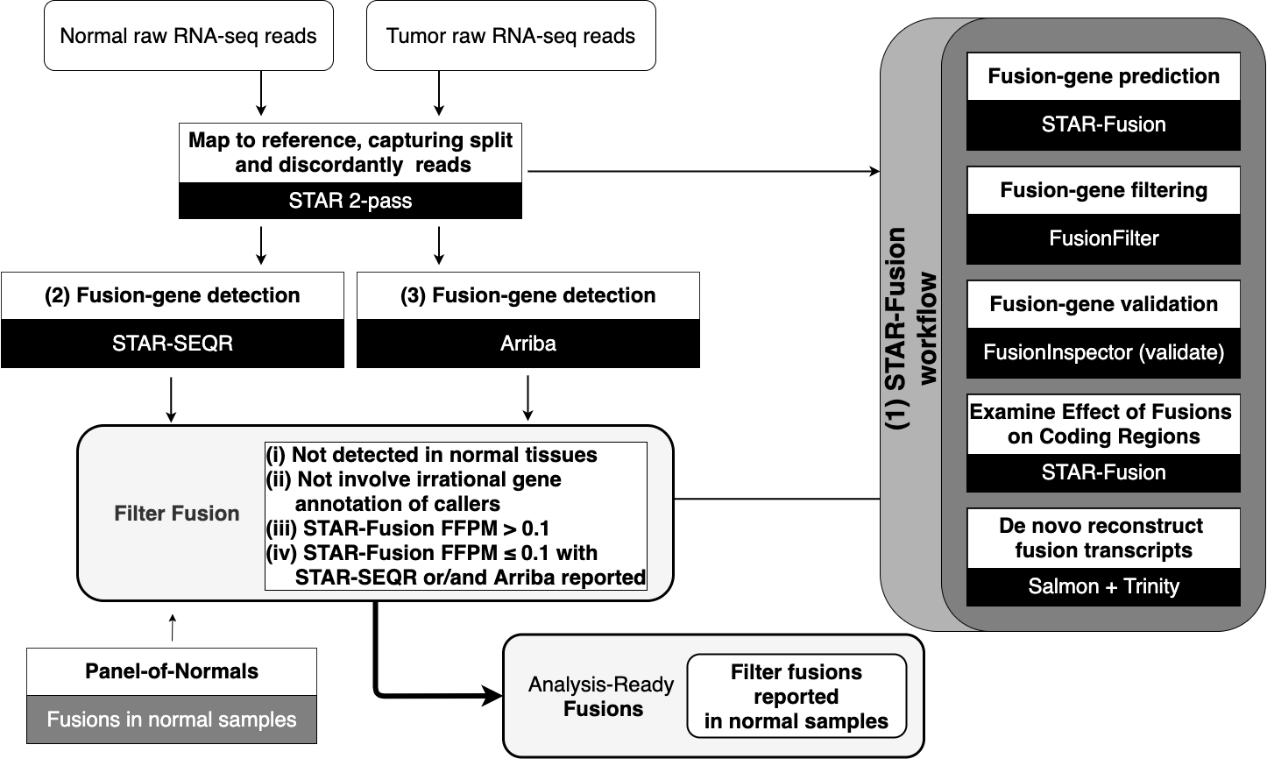


**Figure S10. Workflow for identifying gene fusions.** Arriba: v1.2.0 (<https://github.com/suhrig/arriba/>); STAR: v2.7.2b(32); STAR-SEQR: v0.6.7(<https://github.com/ExpressionAnalysis/STAR-SEQR>); STAR-Fusion (FusionFilter and FusionInspector): v1.8.0(33); Salmon: v0.14.2(54); Trinity: 2.8.5(34). FFPM: fusion fragments per million total fragments.


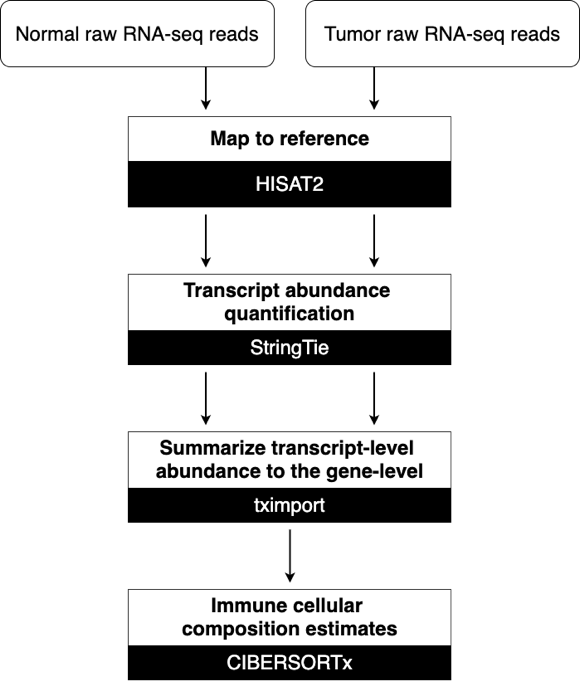


**Figure S11. Workflow for immune cellular composition estimates.** CIBERSORTx: (<https://cibersortx.stanford.edu/index.php>)(38); HISAT2: v2.0.3beta(35); StringTie: v2.0(36); tximport: v1.20.0(37).


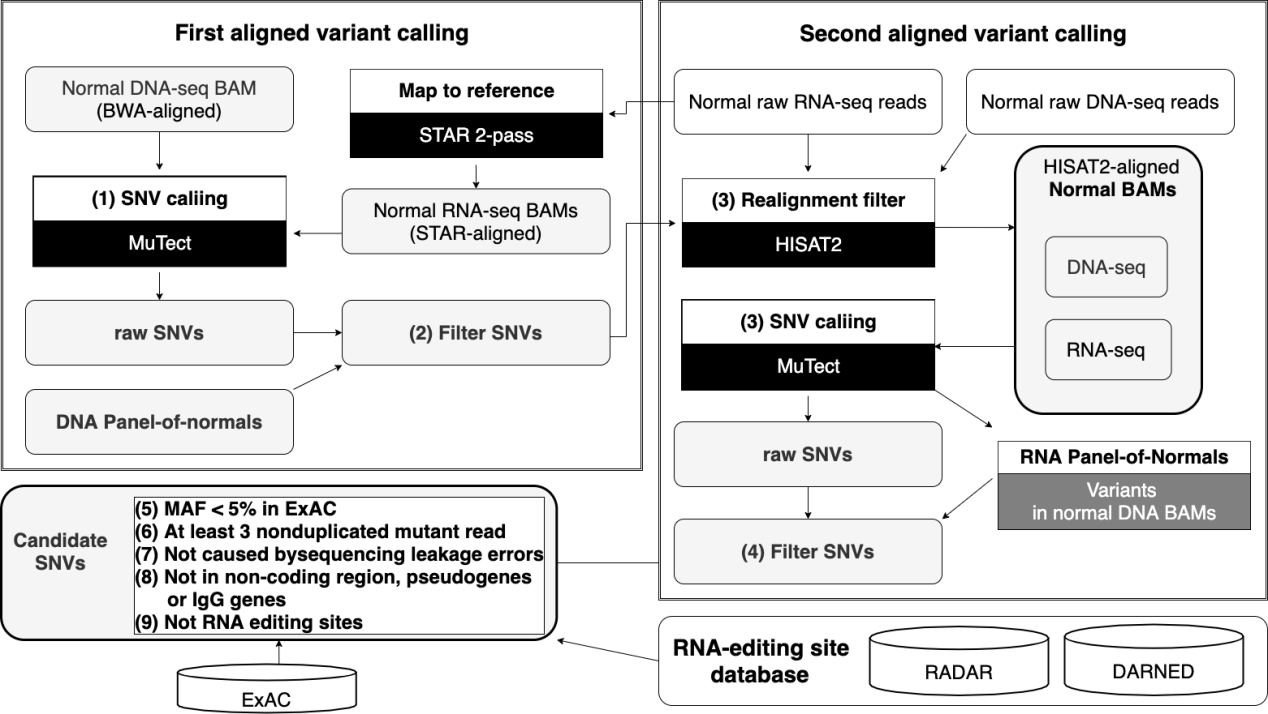


**Figure S12. Workflow for detecting somatic mutations in normal tissues from both RNA and DNA sequencing data using the RNA-Mutect pipeline.** BWA-MEM**:** v0.7.15(12); DARNED(41); ExAC: v0.3.1(18); HISAT2: v2.0.3beta(35); MuTect: v1.1.6^4^; RADAR(40); STAR: v2.6.0c(32). BAM: binary alignment map file; IgG: immunoglobulin G; MAF: mutation annotation format; SNV: single nucleotide variant.


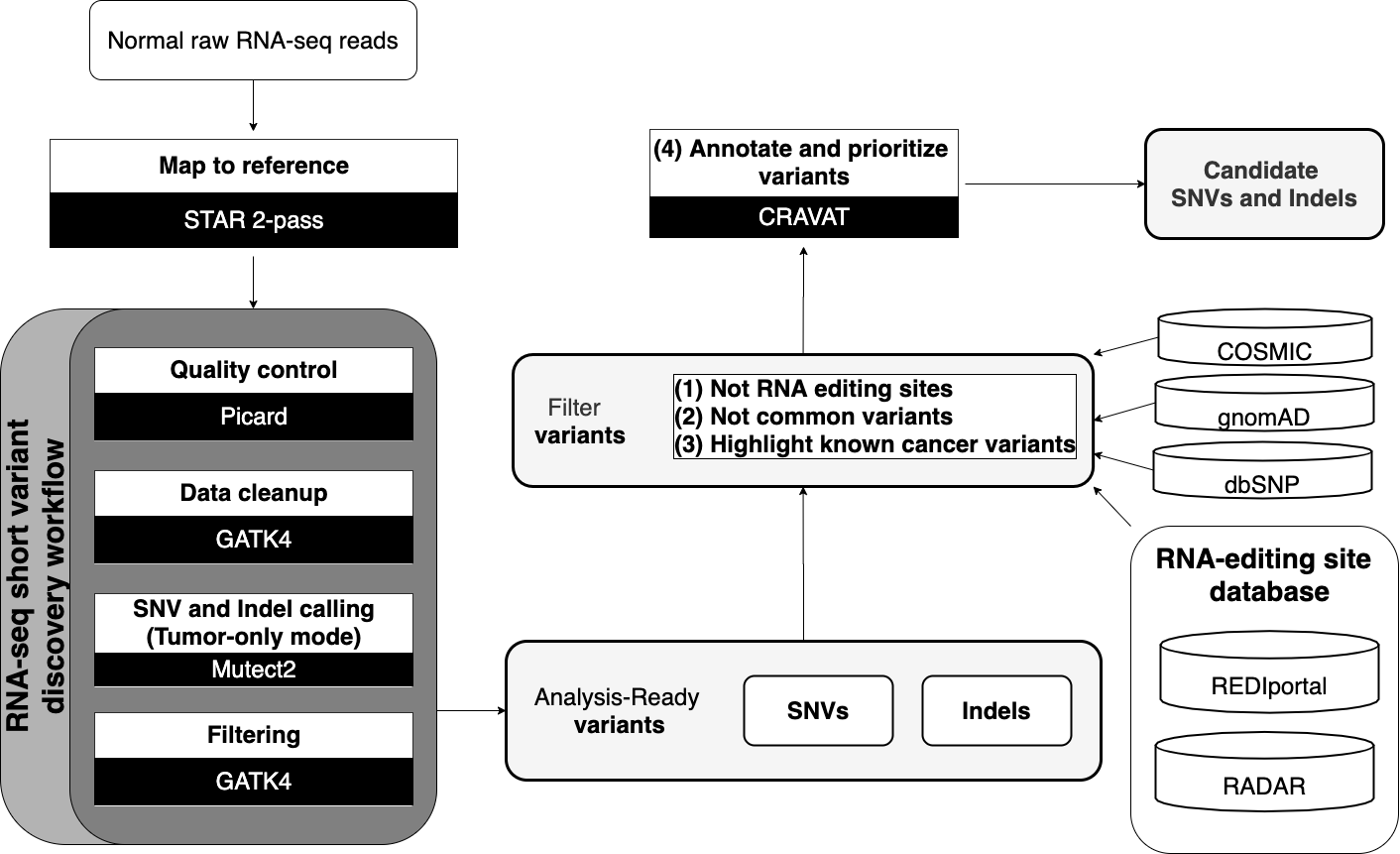


**Figure S13. Workflow for detecting somatic mutations in normal tissues from RNA sequencing data alone using the CTAT-Mutations pipeline.** CRAVAT: v5.2.4(44); COSMIC: v85(16); dbSNP: v151(14); GATK (Mutect2): v4.1.4.1(3, 15); gnomAD(43); Picard: v2.6.0; REDIportal(42); RADAR(40); STAR: v2.7.2b(32). SNV: single nucleotide variant.


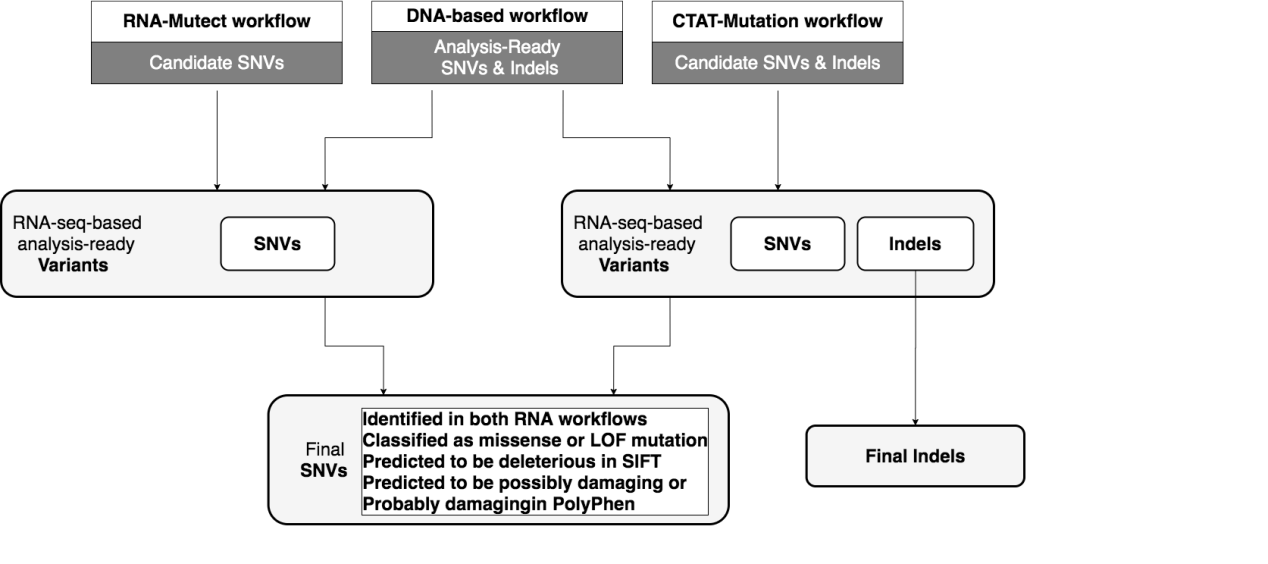


**Figure S14. Workflow for somatic variant calling in normal tissues.** PolyPhen(46); SIFT(45). LOF: loss-of-function; SNV: single nucleotide variant.

# References

1. McLaren W, Gil L, Hunt SE, Riat HS, Ritchie GR, Thormann A, et al. The Ensembl Variant Effect Predictor. *Genome biology* (2016) 17(1):122. Epub 2016/06/09. doi: 10.1186/s13059-016-0974-4. PubMed PMID: 27268795; PubMed Central PMCID: PMCPMC4893825.

2. Tan A, Abecasis GR, Kang HM. Unified representation of genetic variants. *Bioinformatics (Oxford, England)* (2015) 31(13):2202-4. Epub 2015/02/24. doi: 10.1093/bioinformatics/btv112. PubMed PMID: 25701572; PubMed Central PMCID: PMCPMC4481842.

3. DePristo MA, Banks E, Poplin R, Garimella KV, Maguire JR, Hartl C, et al. A framework for variation discovery and genotyping using next-generation DNA sequencing data. *Nature genetics* (2011) 43(5):491-8. Epub 2011/04/12. doi: 10.1038/ng.806. PubMed PMID: 21478889; PubMed Central PMCID: PMCPMC3083463.

4. Rasmussen M, Fenoy E, Harndahl M, Kristensen AB, Nielsen IK, Nielsen M, et al. Pan-Specific Prediction of Peptide-MHC Class I Complex Stability, a Correlate of T Cell Immunogenicity. *Journal of immunology (Baltimore, Md : 1950)* (2016) 197(4):1517-24. Epub 2016/07/13. doi: 10.4049/jimmunol.1600582. PubMed PMID: 27402703; PubMed Central PMCID: PMCPMC4976001.

5. Kesmir C, Nussbaum AK, Schild H, Detours V, Brunak S. Prediction of proteasome cleavage motifs by neural networks. *Protein engineering* (2002) 15(4):287-96. Epub 2002/05/02. doi: 10.1093/protein/15.4.287. PubMed PMID: 11983929.

6. Shen R, Seshan VE. FACETS: allele-specific copy number and clonal heterogeneity analysis tool for high-throughput DNA sequencing. *Nucleic acids research* (2016) 44(16):e131. Epub 2016/06/09. doi: 10.1093/nar/gkw520. PubMed PMID: 27270079; PubMed Central PMCID: PMCPMC5027494.

7. Auton A, Brooks LD, Durbin RM, Garrison EP, Kang HM, Korbel JO, et al. A global reference for human genetic variation. *Nature* (2015) 526(7571):68-74. Epub 2015/10/04. doi: 10.1038/nature15393. PubMed PMID: 26432245; PubMed Central PMCID: PMCPMC4750478.

8. Mermel CH, Schumacher SE, Hill B, Meyerson ML, Beroukhim R, Getz G. GISTIC2.0 facilitates sensitive and confident localization of the targets of focal somatic copy-number alteration in human cancers. *Genome biology* (2011) 12(4):R41. Epub 2011/04/30. doi: 10.1186/gb-2011-12-4-r41. PubMed PMID: 21527027; PubMed Central PMCID: PMCPMC3218867.

9. Chakravarty D, Gao J, Phillips SM, Kundra R, Zhang H, Wang J, et al. OncoKB: A Precision Oncology Knowledge Base. *JCO precision oncology* (2017) 2017. Epub 2017/09/12. doi: 10.1200/po.17.00011. PubMed PMID: 28890946; PubMed Central PMCID: PMCPMC5586540.

10. Sondka Z, Bamford S, Cole CG, Ward SA, Dunham I, Forbes SA. The COSMIC Cancer Gene Census: describing genetic dysfunction across all human cancers. *Nature reviews Cancer* (2018) 18(11):696-705. Epub 2018/10/08. doi: 10.1038/s41568-018-0060-1. PubMed PMID: 30293088.

11. Sanchez-Vega F, Mina M, Armenia J, Chatila WK, Luna A, La KC, et al. Oncogenic Signaling Pathways in The Cancer Genome Atlas. *Cell* (2018) 173(2):321-37.e10. Epub 2018/04/07. doi: 10.1016/j.cell.2018.03.035. PubMed PMID: 29625050; PubMed Central PMCID: PMCPMC6070353.

12. Li H, Durbin R. Fast and accurate short read alignment with Burrows-Wheeler transform. *Bioinformatics (Oxford, England)* (2009) 25(14):1754-60. Epub 2009/05/20. doi: 10.1093/bioinformatics/btp324. PubMed PMID: 19451168; PubMed Central PMCID: PMCPMC2705234.

13. Mills RE, Pittard WS, Mullaney JM, Farooq U, Creasy TH, Mahurkar AA, et al. Natural genetic variation caused by small insertions and deletions in the human genome. *Genome research* (2011) 21(6):830-9. Epub 2011/04/05. doi: 10.1101/gr.115907.110. PubMed PMID: 21460062; PubMed Central PMCID: PMCPMC3106316.

14. Sherry ST, Ward MH, Kholodov M, Baker J, Phan L, Smigielski EM, et al. dbSNP: the NCBI database of genetic variation. *Nucleic acids research* (2001) 29(1):308-11. Epub 2000/01/11. doi: 10.1093/nar/29.1.308. PubMed PMID: 11125122; PubMed Central PMCID: PMCPMC29783.

15. Cibulskis K, Lawrence MS, Carter SL, Sivachenko A, Jaffe D, Sougnez C, et al. Sensitive detection of somatic point mutations in impure and heterogeneous cancer samples. *Nature biotechnology* (2013) 31(3):213-9. Epub 2013/02/12. doi: 10.1038/nbt.2514. PubMed PMID: 23396013; PubMed Central PMCID: PMCPMC3833702.

16. Forbes SA, Beare D, Boutselakis H, Bamford S, Bindal N, Tate J, et al. COSMIC: somatic cancer genetics at high-resolution. *Nucleic acids research* (2017) 45(D1):D777-d83. Epub 2016/12/03. doi: 10.1093/nar/gkw1121. PubMed PMID: 27899578; PubMed Central PMCID: PMCPMC5210583.

17. Costello M, Pugh TJ, Fennell TJ, Stewart C, Lichtenstein L, Meldrim JC, et al. Discovery and characterization of artifactual mutations in deep coverage targeted capture sequencing data due to oxidative DNA damage during sample preparation. *Nucleic acids research* (2013) 41(6):e67. Epub 2013/01/11. doi: 10.1093/nar/gks1443. PubMed PMID: 23303777; PubMed Central PMCID: PMCPMC3616734.

18. Lek M, Karczewski KJ, Minikel EV, Samocha KE, Banks E, Fennell T, et al. Analysis of protein-coding genetic variation in 60,706 humans. *Nature* (2016) 536(7616):285-91. Epub 2016/08/19. doi: 10.1038/nature19057. PubMed PMID: 27535533; PubMed Central PMCID: PMCPMC5018207.

19. Ellrott K, Bailey MH, Saksena G, Covington KR, Kandoth C, Stewart C, et al. Scalable Open Science Approach for Mutation Calling of Tumor Exomes Using Multiple Genomic Pipelines. *Cell systems* (2018) 6(3):271-81.e7. Epub 2018/03/30. doi: 10.1016/j.cels.2018.03.002. PubMed PMID: 29596782; PubMed Central PMCID: PMCPMC6075717.

20. Taylor-Weiner A, Stewart C, Giordano T, Miller M, Rosenberg M, Macbeth A, et al. DeTiN: overcoming tumor-in-normal contamination. *Nature methods* (2018) 15(7):531-4. Epub 2018/06/27. doi: 10.1038/s41592-018-0036-9. PubMed PMID: 29941871; PubMed Central PMCID: PMCPMC6528031.

21. Gao J, Aksoy BA, Dogrusoz U, Dresdner G, Gross B, Sumer SO, et al. Integrative analysis of complex cancer genomics and clinical profiles using the cBioPortal. *Science signaling* (2013) 6(269):pl1. Epub 2013/04/04. doi: 10.1126/scisignal.2004088. PubMed PMID: 23550210; PubMed Central PMCID: PMCPMC4160307.

22. Dees ND, Zhang Q, Kandoth C, Wendl MC, Schierding W, Koboldt DC, et al. MuSiC: identifying mutational significance in cancer genomes. *Genome research* (2012) 22(8):1589-98. Epub 2012/07/05. doi: 10.1101/gr.134635.111. PubMed PMID: 22759861; PubMed Central PMCID: PMCPMC3409272.

23. Martincorena I, Raine KM, Gerstung M, Dawson KJ, Haase K, Van Loo P, et al. Universal Patterns of Selection in Cancer and Somatic Tissues. *Cell* (2017) 171(5):1029-41.e21. Epub 2017/10/24. doi: 10.1016/j.cell.2017.09.042. PubMed PMID: 29056346; PubMed Central PMCID: PMCPMC5720395.

24. Tamborero D, Gonzalez-Perez A, Lopez-Bigas N. OncodriveCLUST: exploiting the positional clustering of somatic mutations to identify cancer genes. *Bioinformatics (Oxford, England)* (2013) 29(18):2238-44. Epub 2013/07/26. doi: 10.1093/bioinformatics/btt395. PubMed PMID: 23884480.

25. Mularoni L, Sabarinathan R, Deu-Pons J, Gonzalez-Perez A, Lopez-Bigas N. OncodriveFML: a general framework to identify coding and non-coding regions with cancer driver mutations. *Genome biology* (2016) 17(1):128. Epub 2016/06/18. doi: 10.1186/s13059-016-0994-0. PubMed PMID: 27311963; PubMed Central PMCID: PMCPMC4910259.

26. Kircher M, Witten DM, Jain P, O'Roak BJ, Cooper GM, Shendure J. A general framework for estimating the relative pathogenicity of human genetic variants. *Nature genetics* (2014) 46(3):310-5. Epub 2014/02/04. doi: 10.1038/ng.2892. PubMed PMID: 24487276; PubMed Central PMCID: PMCPMC3992975.

27. Tokheim CJ, Papadopoulos N, Kinzler KW, Vogelstein B, Karchin R. Evaluating the evaluation of cancer driver genes. *Proceedings of the National Academy of Sciences of the United States of America* (2016) 113(50):14330-5. Epub 2016/12/03. doi: 10.1073/pnas.1616440113. PubMed PMID: 27911828; PubMed Central PMCID: PMCPMC5167163.

28. Vogelstein B, Papadopoulos N, Velculescu VE, Zhou S, Diaz LA, Jr., Kinzler KW. Cancer genome landscapes. *Science (New York, NY)* (2013) 339(6127):1546-58. Epub 2013/03/30. doi: 10.1126/science.1235122. PubMed PMID: 23539594; PubMed Central PMCID: PMCPMC3749880.

29. Bailey MH, Tokheim C, Porta-Pardo E, Sengupta S, Bertrand D, Weerasinghe A, et al. Comprehensive Characterization of Cancer Driver Genes and Mutations. *Cell* (2018) 173(2):371-85.e18. Epub 2018/04/07. doi: 10.1016/j.cell.2018.02.060. PubMed PMID: 29625053; PubMed Central PMCID: PMCPMC6029450.

30. Radovich M, Pickering CR, Felau I, Ha G, Zhang H, Jo H, et al. The Integrated Genomic Landscape of Thymic Epithelial Tumors. *Cancer cell* (2018) 33(2):244-58.e10. Epub 2018/02/14. doi: 10.1016/j.ccell.2018.01.003. PubMed PMID: 29438696; PubMed Central PMCID: PMCPMC5994906.

31. Petrini I, Meltzer PS, Kim IK, Lucchi M, Park KS, Fontanini G, et al. A specific missense mutation in GTF2I occurs at high frequency in thymic epithelial tumors. *Nature genetics* (2014) 46(8):844-9. Epub 2014/07/01. doi: 10.1038/ng.3016. PubMed PMID: 24974848; PubMed Central PMCID: PMCPMC5705185.

32. Dobin A, Davis CA, Schlesinger F, Drenkow J, Zaleski C, Jha S, et al. STAR: ultrafast universal RNA-seq aligner. *Bioinformatics (Oxford, England)* (2013) 29(1):15-21. Epub 2012/10/30. doi: 10.1093/bioinformatics/bts635. PubMed PMID: 23104886; PubMed Central PMCID: PMCPMC3530905.

33. Haas BJ, Dobin A, Li B, Stransky N, Pochet N, Regev A. Accuracy assessment of fusion transcript detection via read-mapping and de novo fusion transcript assembly-based methods. *Genome biology* (2019) 20(1):213. Epub 2019/10/23. doi: 10.1186/s13059-019-1842-9. PubMed PMID: 31639029; PubMed Central PMCID: PMCPMC6802306.

34. Grabherr MG, Haas BJ, Yassour M, Levin JZ, Thompson DA, Amit I, et al. Full-length transcriptome assembly from RNA-Seq data without a reference genome. *Nature biotechnology* (2011) 29(7):644-52. Epub 2011/05/17. doi: 10.1038/nbt.1883. PubMed PMID: 21572440; PubMed Central PMCID: PMCPMC3571712.

35. Kim D, Paggi JM, Park C, Bennett C, Salzberg SL. Graph-based genome alignment and genotyping with HISAT2 and HISAT-genotype. *Nature biotechnology* (2019) 37(8):907-15. Epub 2019/08/04. doi: 10.1038/s41587-019-0201-4. PubMed PMID: 31375807.

36. Kovaka S, Zimin AV, Pertea GM, Razaghi R, Salzberg SL, Pertea M. Transcriptome assembly from long-read RNA-seq alignments with StringTie2. *Genome biology* (2019) 20(1):278. Epub 2019/12/18. doi: 10.1186/s13059-019-1910-1. PubMed PMID: 31842956; PubMed Central PMCID: PMCPMC6912988.

37. Soneson C, Love MI, Robinson MD. Differential analyses for RNA-seq: transcript-level estimates improve gene-level inferences. *F1000Res* (2015) 4:1521. Epub 2016/03/01. doi: 10.12688/f1000research.7563.2. PubMed PMID: 26925227; PubMed Central PMCID: PMCPMC4712774.

38. Newman AM, Steen CB, Liu CL, Gentles AJ, Chaudhuri AA, Scherer F, et al. Determining cell type abundance and expression from bulk tissues with digital cytometry. *Nature biotechnology* (2019) 37(7):773-82. Epub 2019/05/08. doi: 10.1038/s41587-019-0114-2. PubMed PMID: 31061481; PubMed Central PMCID: PMCPMC6610714.

39. Yizhak K, Aguet F, Kim J, Hess JM, Kübler K, Grimsby J, et al. RNA sequence analysis reveals macroscopic somatic clonal expansion across normal tissues. *Science (New York, NY)* (2019) 364(6444). Epub 2019/06/07. doi: 10.1126/science.aaw0726. PubMed PMID: 31171663.

40. Ramaswami G, Li JB. RADAR: a rigorously annotated database of A-to-I RNA editing. *Nucleic acids research* (2014) 42(Database issue):D109-13. Epub 2013/10/29. doi: 10.1093/nar/gkt996. PubMed PMID: 24163250; PubMed Central PMCID: PMCPMC3965033.

41. Kiran A, Baranov PV. DARNED: a DAtabase of RNa EDiting in humans. *Bioinformatics (Oxford, England)* (2010) 26(14):1772-6. Epub 2010/06/16. doi: 10.1093/bioinformatics/btq285. PubMed PMID: 20547637.

42. Picardi E, D'Erchia AM, Lo Giudice C, Pesole G. REDIportal: a comprehensive database of A-to-I RNA editing events in humans. *Nucleic acids research* (2017) 45(D1):D750-d7. Epub 2016/09/03. doi: 10.1093/nar/gkw767. PubMed PMID: 27587585; PubMed Central PMCID: PMCPMC5210607.

43. Karczewski KJ, Francioli LC, Tiao G, Cummings BB, Alföldi J, Wang Q, et al. The mutational constraint spectrum quantified from variation in 141,456 humans. *Nature* (2020) 581(7809):434-43. Epub 2020/05/29. doi: 10.1038/s41586-020-2308-7. PubMed PMID: 32461654.

44. Masica DL, Douville C, Tokheim C, Bhattacharya R, Kim R, Moad K, et al. CRAVAT 4: Cancer-Related Analysis of Variants Toolkit. *Cancer research* (2017) 77(21):e35-e8. Epub 2017/11/03. doi: 10.1158/0008-5472.Can-17-0338. PubMed PMID: 29092935; PubMed Central PMCID: PMCPMC5850945.

45. Ng PC, Henikoff S. SIFT: Predicting amino acid changes that affect protein function. *Nucleic acids research* (2003) 31(13):3812-4. Epub 2003/06/26. doi: 10.1093/nar/gkg509. PubMed PMID: 12824425; PubMed Central PMCID: PMCPMC168916.

46. Adzhubei I, Jordan DM, Sunyaev SR. Predicting functional effect of human missense mutations using PolyPhen-2. *Current protocols in human genetics* (2013) Chapter 7:Unit7.20. Epub 2013/01/15. doi: 10.1002/0471142905.hg0720s76. PubMed PMID: 23315928; PubMed Central PMCID: PMCPMC4480630.

47. Feuerbach L, Sieverling L, Deeg KI, Ginsbach P, Hutter B, Buchhalter I, et al. TelomereHunter - in silico estimation of telomere content and composition from cancer genomes. *BMC bioinformatics* (2019) 20(1):272. Epub 2019/05/30. doi: 10.1186/s12859-019-2851-0. PubMed PMID: 31138115; PubMed Central PMCID: PMCPMC6540518.

48. Niu B, Ye K, Zhang Q, Lu C, Xie M, McLellan MD, et al. MSIsensor: microsatellite instability detection using paired tumor-normal sequence data. *Bioinformatics (Oxford, England)* (2014) 30(7):1015-6. Epub 2013/12/29. doi: 10.1093/bioinformatics/btt755. PubMed PMID: 24371154; PubMed Central PMCID: PMCPMC3967115.

49. Andreatta M, Nielsen M. Gapped sequence alignment using artificial neural networks: application to the MHC class I system. *Bioinformatics (Oxford, England)* (2016) 32(4):511-7. Epub 2015/10/31. doi: 10.1093/bioinformatics/btv639. PubMed PMID: 26515819; PubMed Central PMCID: PMCPMC6402319.

50. Jurtz V, Paul S, Andreatta M, Marcatili P, Peters B, Nielsen M. NetMHCpan-4.0: Improved Peptide-MHC Class I Interaction Predictions Integrating Eluted Ligand and Peptide Binding Affinity Data. *Journal of immunology (Baltimore, Md : 1950)* (2017) 199(9):3360-8. Epub 2017/10/06. doi: 10.4049/jimmunol.1700893. PubMed PMID: 28978689; PubMed Central PMCID: PMCPMC5679736.

51. Szolek A, Schubert B, Mohr C, Sturm M, Feldhahn M, Kohlbacher O. OptiType: precision HLA typing from next-generation sequencing data. *Bioinformatics (Oxford, England)* (2014) 30(23):3310-6. Epub 2014/08/22. doi: 10.1093/bioinformatics/btu548. PubMed PMID: 25143287; PubMed Central PMCID: PMCPMC4441069.

52. Hundal J, Kiwala S, McMichael J, Miller CA, Xia H, Wollam AT, et al. pVACtools: A Computational Toolkit to Identify and Visualize Cancer Neoantigens. *Cancer immunology research* (2020) 8(3):409-20. Epub 2020/01/08. doi: 10.1158/2326-6066.Cir-19-0401. PubMed PMID: 31907209; PubMed Central PMCID: PMCPMC7056579.

53. Murphy C, Elemento O. AGFusion: annotate and visualize gene fusions. *bioRxiv* (2016):080903. doi: 10.1101/080903.

54. Patro R, Duggal G, Love MI, Irizarry RA, Kingsford C. Salmon provides fast and bias-aware quantification of transcript expression. *Nature methods* (2017) 14(4):417-9. Epub 2017/03/07. doi: 10.1038/nmeth.4197. PubMed PMID: 28263959; PubMed Central PMCID: PMCPMC5600148.
